# The Use of Multiscale Molecular Simulations in Understanding a Relationship between the Structure and Function of Biological Systems of the Brain: The Application to Monoamine Oxidase Enzymes

**DOI:** 10.3389/fnins.2016.00327

**Published:** 2016-07-15

**Authors:** Robert Vianello, Carmen Domene, Janez Mavri

**Affiliations:** ^1^Computational Organic Chemistry and Biochemistry Group, Ruđer Bošković InstituteZagreb, Croatia; ^2^Department of Chemistry, King's College LondonLondon, UK; ^3^Chemistry Research Laboratory, University of OxfordOxford, UK; ^4^Department of Computational Biochemistry and Drug Design, National Institute of ChemistryLjubljana, Slovenia

**Keywords:** computational enzymology, molecular dynamics simulation, central nervous system, neural signal transduction, drug design, hydride transfer reaction, neurotransmitter metabolism, multiscale simulations

## Abstract

**HIGHLIGHTS**

- Computational techniques provide accurate descriptions of the structure and dynamics of biological systems, contributing to their understanding at an atomic level.
- Classical MD simulations are a precious computational tool for the processes where no chemical reactions take place.
- QM calculations provide valuable information about the enzyme activity, being able to distinguish among several mechanistic pathways, provided a carefully selected cluster model of the enzyme is considered.
- Multiscale QM/MM simulation is the method of choice for the computational treatment of enzyme reactions offering quantitative agreement with experimentally determined reaction parameters.
- Molecular simulation provide insight into the mechanism of both the catalytic activity and inhibition of monoamine oxidases, thus aiding in the rational design of their inhibitors that are all employed and antidepressants and antiparkinsonian drugs.

Aging society and therewith associated neurodegenerative and neuropsychiatric diseases, including depression, Alzheimer's disease, obsessive disorders, and Parkinson's disease, urgently require novel drug candidates. Targets include monoamine oxidases A and B (MAOs), acetylcholinesterase (AChE), butyrylcholinesterase (BChE), and various receptors and transporters. For rational drug design it is particularly important to combine experimental synthetic, kinetic, toxicological, and pharmacological information with structural and computational work. This paper describes the application of various modern computational biochemistry methods in order to improve the understanding of a relationship between the structure and function of large biological systems including ion channels, transporters, receptors, and metabolic enzymes. The methods covered stem from classical molecular dynamics simulations to understand the physical basis and the time evolution of the structures, to combined QM, and QM/MM approaches to probe the chemical mechanisms of enzymatic activities and their inhibition. As an illustrative example, the later will focus on the monoamine oxidase family of enzymes, which catalyze the degradation of amine neurotransmitters in various parts of the brain, the imbalance of which is associated with the development and progression of a range of neurodegenerative disorders. Inhibitors that act mainly on MAO A are used in the treatment of depression, due to their ability to raise serotonin concentrations, while MAO B inhibitors decrease dopamine degradation and improve motor control in patients with Parkinson disease. Our results give strong support that both MAO isoforms, A and B, operate through the hydride transfer mechanism. Relevance of MAO catalyzed reactions and MAO inhibition in the context of neurodegeneration will be discussed.

## Introduction

Neurodegenerative disorder, such as Alzheimer's disease, Parkinson's disease, and Huntington's disease, is an umbrella term for a range of incurable and debilitating conditions which primarily affect the neurons in the human brain and result in their progressive degeneration and/or death, causing problems with movement (ataxias) or mental functioning (dementias). Although perceived as the diseases of the elderly, these disorders can have a much earlier onset, starting even before the age of 40, leading to long-term treatment and substantial financial burden for the public healthcare systems. Two recent landmark reports (Gustavsson et al., 2011; Wittchen et al., 2011), conducted within EU–27 countries plus three allied countries (Norway, Iceland, Switzerland), providing the most complete picture ever of these disorders in Europe, estimated that the societal burden of brain disorders is immense: over 160 million people are affected, more than 36% of the total population, while the costs are equally disturbing amounting to a total of €798 billion per year in 2010—one third of all health related expenses—higher than for heart diseases, cancer, and diabetes combined. A breakdown of the nature of the costs shows that just over one third are direct healthcare costs, a quarter direct non-medical costs and the remaining 40% indirect costs associated with loss of productivity. In spite of this, there is at present no effective treatment able to slow down or stop the deterioration of brain function. Early diagnosis is close to impossible, and because it often occurs too late, treatment to mitigate the effects of the illness remains limited. It is, therefore, highly desirable to understand those complex processes at the molecular level in order to pave way toward prevention, diagnosis, and treatment.

Biogenic amines are a large group of naturally occurring biologically active compounds, most of which act as neurotransmitters—endogenous chemicals that allow the transmission of signals from a neuron to target cells across synapses. Neurotransmitters can be excitatory, where there is a direct communication between the presynaptic neuron and postsynaptic neuron through the synaptic gap, or neuromodulators, where the mode of transmission includes diffusion transport over large distances in the central nervous system. There are five established amine neurotransmitters: the three catecholamines (dopamine, noradrenaline, and adrenaline), histamine, and serotonin. These substances are active in regulating many centrally mediated body functions, including behavioral, cognitive, motor and endocrine processes, and can cause adverse symptoms when they are out of balance (Di Giovanni et al., 2008). Despite immense functional importance, we are currently far from understanding the complex cellular and molecular actions of biogenic amines, the timing of their release and the full spectrum of processes that are influenced by them. An important prerequisite to fully realize the actions of biogenic amines is to study, at the molecular level, the mechanisms of both the catalytic activity and inhibition of their metabolic enzymes, and the way these small molecules interact with larger systems such as transporters and receptors, which all represent the starting points for development of tools to diagnose and drugs to treat specific clusters of symptoms of neurodegenerative disorders.

Brain monoaminergic systems have been extensively implicated in the etiology and course of various neurodegenerative disorders, causing problems with movement (ataxias) or mental functioning (dementias; Di Giovanni et al., 2008; Ramsay, 2012). In spite of this, there is, at present, no effective treatment able to substantially slow down or even stop the deterioration of neurons resulting in impaired brain function. All drugs employed nowadays exhibit a range of adverse effects, and drugs tend to address only symptoms rather than the causes of the dysfunction. Early diagnosis is close to impossible and, because it often occurs too late, treatment to mitigate the effects of the illness remains limited. In view of this background, the development of novel compounds for psychiatric manifestations in neurodegenerative disorders is not only of scientific interest to advance understanding of the brain at a systems level, but also fundamental to improving the management of symptoms, the therapeutic compliance and the quality of life of patients.

The present paper focuses on the use of modern molecular dynamics and multi-scale methods of computational biochemistry—from classical molecular dynamics simulations to quantum mechanical (QM) and combined quantum mechanical/molecular mechanical (QM/MM) approaches within the Empirical Valence Bond framework—in order to aid in understanding of a close relationship between the structure and function of some biological systems of the brain with particular focus on a common metabolic enzyme, monoamine oxidase (MAO), responsible for regulating the concentration of biogenic, and dietary amines in various parts of the body. Elucidating the precise atomistic details about the catalytic activity of MAO enzymes is of paramount importance for understanding the chemistry of neurodegeneration on molecular level. The knowledge gained in this area would facilitate research on other members of the large family of flavoenzymes and would, in addition to general biochemistry and physiology, be of significant value for designing and preparing novel effective MAO inhibitors as transition state analogs, which are potential clinical drugs for the treatment of depression, Parkinson, and Alzheimer diseases (Youdim et al., 2006).

## Classical molecular dynamics of proteins

Molecular dynamics simulation (MD) is a powerful computational technique that provides accurate descriptions of the structure and dynamics of biological systems, contributing to their understanding at an atomic level. In MD simulations, the motion of interacting particles is calculated by integrating Newton's equations of motion. The potential energy of the system and the forces, derived from the negative gradient of the potential with respect to displacements in a specified direction, are used to forecast the time evolution of the system in the form of a trajectory. Equilibrium quantities are then calculated using statistical mechanics by averaging over trajectories of sufficient length which would have sampled a representative ensemble of the state of the system. The potential energies can be obtained by either classical or quantum mechanical methods, with the former predominant due to reduced computational expense of utilizing empirical force fields. A force field refers to the functional form and parameter sets used to calculate the potential energy of a system of particles classically. A wide variety of force fields for biological molecules are available including, but not limited to, CHARMM (Chemistry at Harvard Molecular Mechanics; Brooks et al., 1983), AMBER (Assisted Model Building with Energy Requirement; Cornell et al., 1995), and OPLS (Optimized Potentials for Liquid Simulations; Jorgensen and Tirado-Rives, 1988). Each varies in their functional form and parameters therein, which are generally obtained to provide a suitable reproduction of experimental and/or quantum mechanical data. In classical potentials, atoms are considered as spheres with a particular mass and associated charge interlinked by springs that model the bonds. Atomic motions are evaluated using classical mechanics under the Born-Oppenheimer approximation where it is assumed that the motion of atomic nuclei and electrons in a molecule can be separated as a result of the vast difference in mass between electrons and nuclei. Electrons are said to adjust “instantaneously” to changes in the nuclear positions, and they can be ignored when solving the equations of motion. For this reason, the analytic expression that represents the energy of a system described by classical potentials is composed solely by inter- and intra-molecular contributions to the energy function. The most time-consuming part of the simulation is the calculation of the non-bonded interactions. A general functional form includes bond stretching, angle bending, bond rotations, and non-bonded terms. In general, all these interactions are calculated between pairs of atoms neglecting the many-body nature of some of the interactions. This many-body nature refers to the fact that the motion of every single particle influences and depends on the motion of the surrounding particles which would require coupled equations to describe the dynamics of the system. Classical force fields typically consider effective values of electronic polarization which is a significant limitation inherent to all additive force fields, although significant efforts to develop polarizable force fields for biological molecules have resulted in several schemes including the fluctuating charge model, the induced dipole model, and the Drude oscillator approach (Lamoureux et al., 2003; Patel and Brooks, 2004; Patel et al., 2004; Shi et al., 2013). A large majority of biomolecular simulation is currently performed by effectively polarized force fields. In addition, in order to describe chemical reactions, bond breaking or forming, nuclear effects, etc., a quantum formulation is required such as the one described. In systems with a few atoms, solutions of the aforementioned equation of motions can be achieved analytically. However, in larger systems, the subsistence of a continuous potential instigates a many body problem for force evaluations, rendering analytic solutions unattainable. Under these circumstances, finite difference methods are employed and forces are assessed at discrete intervals.

Although the basic idea behind classical molecular dynamics appears to be rather simple, in practice there are many complications and one has to be careful at setting the initial conditions, analyzing in a systematic way the influence of simulation protocols to ensure reliability, choosing appropriate algorithms or storing, and analyzing the huge amount of data generated. Among many applications, classical MD has become an established tool to identify putative binding sites, and consequentially establish how drugs function on a molecular level, to study transport in proteins, to rationalize conformational changes or to study aggregation and recognition. Nonetheless, the high computational expense of atomistic MD simulations for large biological systems remains a significant limitation. Many biological phenomena occur on extended timescales which are generally unattainable by classical MD and alternative approaches to accelerate sampling have been developed. Coarse-grained or reduced representations of molecules where a group of atoms is treated as a single entity or a “bead” are one of such approaches. By employing classical MD simulations using coarse-grained models, larger systems, and longer timescales can be achieved. In addition, several algorithms also exist to accelerate sampling along a pre-defined set of reaction coordinates and estimate the potential of mean force of a process. Enhanced sampling algorithms are crucial to investigate processes that involve overcoming an energetic barrier or exceeding the microsecond timescale, whilst maintaining full atomic detail or when a substantial system size is required even when considering a coarse-grained representation. Among these techniques are thermodynamic integration, umbrella sampling, metadynamics, adaptive biasing force, steered MD, and many other variants.

With the relentless development of computational algorithms together with progress in the experimental determination of three-dimensional structures of membrane proteins and the increasing speed and availability of supercomputers, it is now possible to investigate an immense range of biological phenomenon using MD simulations.

## Quantum mechanical treatment of the chemical reactivity

The past century has seen substantial advances, both in computational techniques and in our understanding of how enzymes really work. The use of quantum chemical methods to address enzymatic reaction mechanisms has become a booming area in enzymology (Ramos and Fernandes, 2008; Lonsdale et al., 2010; Shaik et al., 2010; Carvalho et al., 2014). Nevertheless, studying an enzyme at atomic resolution is a computationally demanding task. In this case, the complexity of the system prevents resorting to pure quantum-mechanical (QM) methods to treat the entire system, while the phenomena under study cannot be accurately represented by molecular-mechanical (MM) methods (since MM methods are in their traditional functional form unable to describe chemical reactions). Currently, there are two popular approaches to describe enzymatic processes: the quantum mechanics-only (QM-only) (Himo, 2006; Siegbahn and Borowski, 2006; Siegbahn and Himo, 2011), which uses a small but carefully selected cluster model of the active site, and the multiscale quantum mechanics/molecular mechanics (QM/MM) method (Senn and Thiel, 2009; van der Kamp and Mulholland, 2013), which employs a layer-based approach using the entire protein. Both methods have been successfully applied to the study of various classes of enzymes, and in many cases similar results and conclusions have been obtained (Ramos and Fernandes, 2008; Shaik et al., 2010).

In the QM-only approach, commonly also called the cluster approach (Siegbahn and Borowski, 2006), a model of the active site is designed on the basis of available crystal structures. The basic idea of this approach for modeling enzyme active sites and reaction mechanisms is to cut out a relatively small but well-chosen part of the enzyme and treat it with as accurate quantum chemical methods as possible. The model should be a good representation of the whole enzyme, and should behave and react like the real system. Hybrid density functional theory (DFT) methods are most frequently used for the calculation of the geometries and energies of all stationary points along the reaction pathways. The missing steric and electrostatic effects from the remaining part of the protein are considered by two simple procedures. The steric effects imposed by the protein matrix are taken into account by applying position constraints to certain key atoms at the periphery of the cluster model, while electrostatic effects are modeled by the dielectric cavity method, usually with a dielectric constant of four. In this respect the experimental structure is included in the calculation. Recent work even demonstrated that the solvation effects saturate with increasing the size of a cluster and that the particular choice of dielectric constant is then no longer of much concern (Liao et al., 2011).

The first application of the cluster model on an enzyme reaction mechanism using a high accuracy electronic structure method was done for methane monooxygenase in 1997 (Siegbahn and Crabtree, 1997). In the past 20 years, the cluster approach has gradually developed to become more robust and accurate, a work that is still continuing. For example, different models of the amino acids were tested (Siegbahn, 2001), and also how second-shell amino acids should be handled (Pelmenschikov et al., 2002). As a result of the accelerating computer power, active site models have become increasingly larger, which has enabled the modeling of evermore complex problems. At present, models with up to 200 atoms are often used, which should yield higher accuracy but has also introduced several new problems in the modeling, for example, a large number of local geometric minima. These problems are minimized by trying to limit the size of the model and include only those residues that are found to have significant effects. When this is successful, the approach is extremely powerful and yields a wealth of new information. On the basis of extensive calculations for a large number of enzymes, the error of the cluster approach for modeling metalloenzymes has been assessed by Siegbahn and co-workers to be < 5 kcal/mol (Siegbahn, 2006).

## Multiscale simulation on the QM/MM level

Despite a tremendous increase in computer power, including the availability of tailor-made massively parallelized computer architectures, together with specialized and more efficient computer algorithms, it is still impossible to rigorously treat chemical reactivity in enzymes and solution describing the entire system at the quantum chemical level. Two key underlying issues for a correct description of reactions and processes with chemical character are: (i) an accurate and computationally efficient description of the bond breaking/forming processes, and (ii) proper modeling of the complex environment of the reaction, which involves efficient thermal averaging of the energy landscape. For converged calculations of free energy profiles for a typical enzyme reaction with applied position restraints for reactive species, it is necessary to perform at least a nanosecond trajectory, what requires evaluation of forces and energies for a million configurations. The use of molecular mechanics (MM) approaches, which are based on classical potentials, is extremely helpful, as they allow inclusion of environmental effects (either solvent molecules or enzymes) in a cost-efficient way. However, traditional MM force field functional forms are unable to describe changes in the electronic structure associated with making and breaking chemical bonds of a system undergoing a chemical reaction.

A solution to these challenges is the use of multiscale approaches, in which the interesting part of the system (usually the reactive atoms plus some additional environment) is described at the electronic level by high-level QM models, while the rest of the system is represented by empirical force fields (or by a lower-level QM method). Multiscale approaches have now become established *state-of-the-art* computational techniques for the modeling of chemical reactions in the condensed phase, including complex processes in organic chemistry, biochemistry, and heterogeneous catalysis, among others. Over the past decade, a number of so-called combined quantum mechanical and molecular mechanical (QM/MM) methods have been implemented, using different approximations and interaction schemes. The award of the 2013 Nobel Prize in Chemistry to Martin Karplus, Michael Levitt, and Arieh Warshel for “the development of multiscale models for complex chemical systems” has demonstrated how mature and highly important multiscale simulations for enzymology and drug design are.

*Ab initio* or DFT QM/MM methods are still computationally very demanding since they do not allow for well-converged reaction free energy profiles. The Empirical Valence Bond (EVB) approach introduced by Warshel and Levitt (1976) was the first QM/MM method, and after nearly four decades it remains the most practical approach in computational enzymology and the computational treatment of chemical reactivity in polar solutions in general. In their seminal work *Theoretical Study of Enzymatic Reactions: Dielectric, Electrostatic, and Steric Stabilization of the Carbonium Ion in the Reaction of Lysozyme* (Warshel and Levitt, 1976), Warshel and Levitt introduced all of the basic concepts of the QM/MM methodology, including the partitioning of the system, the form of the potential energy function, and the interactions between the QM and MM parts. The rest of this section will be devoted to the description of EVB that provides a powerful way to connect classical concepts of physical organic chemistry to the study of chemical reactions. On purpose, most of the mathematical formulation is not shown, and the interested reader is referred to review articles describing progress in EVB theory and implementation (Olsson et al., 2006; Kamerlin and Warshel, 2011a,b).

As within a standard Valence Bonds (VB) framework, EVB uses a set of VB configurations, which can involve covalent, ionic, or a mixture of bonding types, to describe the reactive system participating in the chemical reaction. However, in this case, each VB state corresponds to different bonding patterns of key essential energy minima (reactants, products, and any intermediates) along the postulated reaction coordinate. Energy of covalent bonds is described by Morse functions that allow for bond breaking and making, while bond angle and dihedral angle terms are functionally identical to other force fields. For molecular simulation of a simple S_N_2 reaction with the EVB method with graphical interface visit http://www.ki.si/L01/EVB.

Most enzymatic reactions involve high barriers that cannot be sampled directly by molecular dynamics. In order to calculate reaction energy profiles in terms of free energy, it is necessary to proceed with biased sampling in conjunction with a special method for the free energy calculation. The strategy for this involves the free-energy perturbation (FEP) approach and the so-called umbrella sampling (US) procedure. The FEP approach is based on gradual transformation between the reactant and the product state using the coupling parameter, λ. At each step, a molecular dynamics (MD) simulation is performed with fixed λ. This technique ensures that the system explores areas of the phase space that would not be accessible otherwise in real time, due to the high potential energy (i.e., around the transition state).

The reaction barrier, ΔG^‡^, extracted from the computed free energy profile is directly related to the rate constant k as k = $\frac{{\text{K}}_{\text{B}}\text{T}}{\text{h}}\text{exp}\left(-\frac{\Delta {\text{G}}^{‡}}{{\text{K}}_{\text{B}}\text{T}}\right)$, where T is the absolute temperature while h and k_B_ are Planck and Boltzmann constant, respectively. Therefore, once the free energy profiles are computed, they can be trivially converted into reaction rates. The applied procedure proved to be computationally robust and allows for practical calculation of the reaction rates. Currently, many research groups are actively developing and applying different approaches based on the EVB philosophy. These recent developments, which include new methodologies and program packages, have greatly expanded the scope of problems that can be studied with EVB, enabling applications from small to large-scale molecular systems. Still, computational enzymology remains the most important field where EVB is applied.

## Examples of classical molecular dynamics of membrane proteins

Neurotransmitters such as dopamine and serotonin play a central role in the pathophysiology of major neuropsychiatric illnesses, such as anxiety and mood disorders, schizophrenia, autism spectrum disorders, Parkinson's disease, epilepsy, and dementias. Neurotransmitter-binding proteins such as receptors, transporters, and common metabolic enzymes are the starting points for development of tools to diagnose and drugs to treat specific clusters of symptoms. How these proteins function on a molecular level, remains largely unresolved. A fuller understanding is steadily emerging due to the increasing availability of three-dimensional structures of membrane proteins, in combination with computational methodologies, such as molecular dynamics simulations. In this context, computational labs have focused their efforts in studying at atomistic level how membrane proteins work probing events such as selectivity, permeation, gating, or the conformational changes associated with these processes, and more recently, on studies illustrating protein-lipid interactions (Domene, 2007; Domene and Furini, 2009; Illingworth and Domene, 2009; Furini and Domene, 2013; Ingolfsson et al., 2014). For instance, the composition of biological cell membranes is integral in controlling the structure, and subsequently function, of membrane proteins. Cholesterol is an essential component of the plasma membrane, with concentrations of over 30% in some tissues (Lange, 1991). It has been identified to be extremely influential in the functioning of G-protein couple receptors (GPCR's) such as the serotonin_1A_ receptor, β2-adrenergic receptor and the rhodopsin receptor (Burger et al., 2000; Pucadyil and Chattopadhyay, 2006; Paila and Chattopadhyay, 2010; Oates and Watts, 2011; Jafurulla and Chattopadhyay, 2013). However, the mechanism by which cholesterol modulates protein structure and function is largely unknown. Various theories have been proposed throughout the literature suggesting that cholesterol acts by either varying the physical properties of the membrane, binding directly to specific sites on the protein surface, or facilitating interactions with a third party which biases the activation state. Indeed, these effects may not be mutually exclusive but act cooperatively. Crystallographic data of GPCR's in complex with cholesterol molecules, such as the β2-adrenergic receptor (Cherezov et al., 2007; Hanson et al., 2008), β1-adrenergic receptor (Warne et al., 2011), A_2A_-adenosine receptor (Liu et al., 2012), 5-HT_2B_-receptor (Wang et al., 2013), and metabotropic glutamate type 1 receptor (Wu et al., 2014), has led to the identification of specific cholesterol interaction sites. Elucidation of the β2-adrenergic receptor (Hanson et al., 2008) structure established the cholesterol consensus motif (CCM), a groove region between helices II and VII constituted of highly conserved residues and sequence analysis of several GPCR's identified additional putative binding sites (Jafurulla et al., 2011), the cholesterol recognition/interaction amino acid consensus (CRAC) motif (Li and Papadopoulos, 1998) and the inverted form of the CRAC motif (Baier et al., 2011), termed the CARC motif. However, the affinity of cholesterol for these sites and the influence on the functional state of GPCR's has not been widely categorized and thus MD simulations are underway to investigate the interactions of GPCR's and cholesterol.

Likewise, ion channels are a large and bio-medically important family of membrane proteins that constitute significant drug targets. They govern the electrical properties of the membranes of excitable cells such as neurons or myocytes by allowing thousands of millions of ions to diffuse down their electrochemical gradient across the membrane. Channels do not stay open all of the time but they are “gated” by changes in voltage across the membrane, changes in the pH, the binding of small molecules, etc. This process is called “gating” and it operates via conformational changes. One of the keys to rationalize the way drugs modulate ion channels is to understand the ability of such small molecules to access their respective binding sites, from which they can exert an activating or inhibitory effect. Drug access depends both on the target conformational state as well as on the real-time dynamics of the residues lining the fenestrations and entry tunnels of the target. Although many computational studies have probed the mechanisms of selectivity and gating as well as the energetics of ion permeation, few have focused on investigating the existence and characteristics of cavities in ion channels through which drugs can exert their action. For example, a recent study in our lab explored the presence, structure and conformational dynamics of transmembrane fenestrations accessible by drugs in potassium channels (Jorgensen et al., 2015). Molecular dynamics simulation trajectories were analyzed from three potassium channels one from a different family, a voltage-gated channel, an inward rectifying channel, and a two-pore domain one. Four lateral fenestrations across the range of potassium channels studied were identified and using structural and sequence alignment and analysis was carried out to characterize the similarities and differences among the K^+^-channel subtypes considered. The study rendered a framework for rationalizing the differential response of potassium channels to drugs targeting the transmembrane domain of these proteins and provided insight about the potential role of lateral fenestrations in K^+^-channels for drug delivery and for designing single and multi-target drugs with improved selectivity.

Another example where MD simulations have recently contributed to the atomistic understanding of a membrane protein is that of the transient receptor potential (TRP) ion channels. The three-dimensional structure of the vanilloid receptor 1 or TRPV1, and TRPV2 were recently determined by single particle electron cryo-microscopy, providing the opportunity to explore ionic conduction in TRP channels at atomic detail (Liao et al., 2013; Zubcevic et al., 2016). They constitute an extensive family of cation channels involved in the ability of organisms to detect noxious mechanical, thermal, and chemical stimuli that give rise to the perception of pain. TRPV1 is the main representative of a subfamily of thermosensitive TRP channels that enable a sensation of scalding heat and pain. Additionally, tissue damage and inflammation products modulate the channel by decreasing its thermal activation threshold (~43°C). This feature makes the TRPV1 channel an essential player in the molecular mechanisms responsible for injury-related hyperalgesia and pain (Brederson et al., 2013; Julius, 2013). In addition to temperature greater than 43°C and acidic conditions, TRPV1 is also activated by capsaicin and allyl isothiocyanate, the irritating chemical in hot chili peppers and the pungent compound in mustard and wasabi, respectively (Everaerts et al., 2011; Julius, 2013). MD simulations using the TRPV1 channel revealed the presence of three main binding sites for cations in the transmembrane domain that were not appreciable in the cryo-EM structure (Darre et al., 2015). Two rings of acidic residues are disposed at the extracellular side of the conduction path generating an electrostatic attraction to cations and a delineating the first binding site. A second binding site was found at the intracellular side of the selectivity filter, and a further site in the central water-filled cavity analogous to those observed in other ion channels. Simulations evidenced the flexibility of the selectivity filter, the area of the protein that discriminates the type of ions that permeates the pore that responds to the type of permeating ions by adjusting the global symmetry of this area. A comprehensive picture of the thermodynamics governing the binding of capsaicin to the TRPV1 channel was also accomplished (Darre and Domene, 2015) by combining molecular docking, unbiased MD simulations, and free energy methods in good agreement with earlier experimental reports. These exploratory calculations have provided an early glimpse of the amino acids engaged in favorable capsaicin–channel interactions defining the key structural determinants of the TRPV1 vanilloid binding site.

Ion channels modulate electrical signals across cell membranes and therefore are key in a myriad of physiological processes. Establishing the mechanisms of selectivity, conduction, and gating is central in biomedical sciences studies in order to establish the connection between structure and function. For many years, molecular dynamics simulations have been used to explore these relationships, and it is likely that they will continue to do so in the future, where the challenges lie initially in tackling systems where the realism of the biological environment is improved and subsequently in simulating higher and more complex levels of organization in living systems.

## Pharmacology of monoamine oxidases

**Scheme 1:**
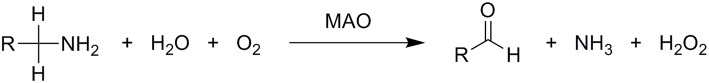
**Overall oxidative deamination of biogenic and dietary amines catalyzed by the MAO enzyme**. Amines are enzymatically converted to the corresponding imines, which leave the MAO active site and are non-enzymatically hydrolyzed to aldehydes.

Monoamine oxidases are mitochondrial outer membrane-bound enzymes that catalyze the oxidative deamination of a broad range of biogenic and dietary amines into their corresponding imines, thus playing a critical role in the degradation of monoamine neurotransmitters in the central and peripheral nervous systems. They contain the covalently bound cofactor flavin adenine dinucleotide (FAD) and are, thus, classified as flavoproteins. Formed imines leave the active site and are then non-enzymatically hydrolyzed to the final carbonyl compounds and ammonia. The enzyme itself is regenerated to its active form by molecular oxygen, O_2_, which is in turn reduced to hydrogen peroxide, H_2_O_2_, according to the overall equation (Scheme 1):

The aldehyde intermediate is rapidly metabolized, usually by oxidation via the enzyme aldehyde dehydrogenase to the corresponding carboxylic acid, or is reduced in some circumstances to the alcohol or glycol by the enzyme aldehyde reductase. MAOs operate using the FAD cofactor, which is, in contrast to the majority of other flavoenzymes, covalently bound to a cysteine through an 8α-thioether linkage (Miller and Edmondson, 1999a). During the catalytic reaction, FAD is reduced to FADH_2_ by accepting two protons and two electrons from the substrate. Although having around 70% sequence identity and a conserved pentapeptidic sequence (Ser-Gly-Gly-Cys-Tyr) that binds the identical FAD cofactor (Klinman, 2007), both the A and the B isoforms of MAO differ on the basis of their substrate affinities and inhibitor sensitivities. However, it is assumed that they act by the same mechanism. In humans, MAO A predominates in the gastrointestinal tract, placenta, and heart, whereas MAO B predominates in platelets and glial cells in the brain. Both are found in liver where biogenic amines are rapidly metabolized and excreted. The primary role of MAO in the gastrointestinal tract is removal of monoamines absorbed by food in order to prevent their uncontrolled interaction with the receptors. It is interesting to mention that the required time for MAO synthesis in the gastrointestinal tract is 2–3 days, while in the central nervous system it is about 2–3 weeks. Under normal physiologic conditions, noradrenalin and serotonin are the preferred substrates of MAO A, while dopamine and β-phenylethylamine are the preferred substrates of MAO B, while both isoforms metabolize dopamine, albeit with differing kinetic parameters. Inhibitors that mainly act on MAO A are used in the treatment of depression, due to their ability to raise serotonin concentrations. In contrast, MAO B inhibitors decrease dopamine degradation and improve motor control in patients with Parkinson disease. Inhibition of MAOs has a notable neuroprotective effect, since MAO catalyzed reactions yield neurotoxic products such as hydrogen peroxide and aldehydes (Gadda, 2012; Edmondson, 2014; Pavlin et al., 2016). The cloning of two separate cDNAs encoding two isoforms of MAO by Shih and co-workers (Bach et al., 1988) provided the basis for a range of important discoveries, thereby allowing the elucidation of their biological roles and development of inhibitors. However, despite tremendous research efforts devoted to MAOs over several decades, neither the catalytic nor the inhibition mechanisms of MAO have been unambiguously established.

## Structures of MAO A and MAO B isoforms

Structures of MAO A and MAO B appeared relatively late and the main obstacle was the protein crystallization. They are trans-membrane proteins and their crystallization represents a very demanding task. After successful heterologous over-expression and purification of recombinant human MAO in yeast or *P. Pastoris* (Newton-Vinson et al., 2000; Li et al., 2002), the three-dimensional structures of human MAO A (Binda et al., 2002; De Colibus et al., 2005) and MAO B (Son et al., 2008) have been solved at a resolution of 2.2 Å and 1.65 Å, respectively. The X–ray structures of both human MAO, A and B, showed that the active-site cavities extend from the flavin-binding site at the core to the surface of the protein. The FAD cofactor binding site is highly conserved between the two enzymes, but several details in the substrate-binding site show major differences. It was hypothesized that substrate preferences and inhibitor specificities are attributed to differences between Phe208–Ile335 in MAO A and Ile199–Tyr326 in MAO B (Li et al., 2006). The covalently-bound FAD lies at the end of a long tunnel leading from the outside of the protein close to the membrane surface. The tunnel is generally considered to be hydrophobic, ending in an aromatic cage near the flavin where two tyrosines align the substrate toward the C4–N5 region of the flavin (Li et al., 2006; Akyuz et al., 2007). The influence of the catalytic site tyrosines (Tyr398 and Tyr435 in MAO B) has been investigated in careful kinetic investigation of several mutants. Although neither is essential to catalysis in terms of complete loss of activity, the affinity for and turnover of substrates is altered in the mutant enzymes. For example, the *K*_m_ for benzylamine increases by more than 10-fold in the Tyr435Phe mutant (Li et al., 2006). The evidence reveals a clear role for these tyrosines in aligning the substrate correctly for catalysis, perpendicular to the N5 and on the *re* face of the isoalloxazine ring. These tyrosines also exert a dipole effect on the substrate that can make the amine more susceptible to oxidation. The key features of substrate position in the active site are proximity and orientation relative to the N5–C4a region of the flavin ring. All these features result in changed activation free energy and therewith associated rate constants on the point mutations.

At this point it is worth to emphasize that availability of the high-resolution MAO structures represents crucial information for the multiscale simulation of the reactive step and rational design of both irreversible and reversible inhibitors.

## MAO preorganized electrostatics is reflected in the p*K*_a_ values

Computational studies gave clear evidence that the enzyme active sites provide specific polar environments that do not resemble the gas phase or simple solvent. The enzyme environment is designed for the electrostatic stabilization of transition states, enabling solvation higher than in water. Therefore, the bulk of the enzyme catalytic power originates from the electrostatic preorganization of its active sites. Essentially, solvent molecules must reorient during the reaction due to polarization induced by a changing charge distribution, while this energetic penalty is much smaller in enzymes, as they provide an electrostatic environment that has evolved to require much less reorganization. A change in the protonation states of ionizable residues results in an altered electrostatic potential pattern in the enzyme, which gives rise to altered activation free energies and manifests as pH dependence of the reaction rate. Electrostatic potential of the enzyme active site is, by definition, a fluctuating scalar field and cannot be directly measured experimentally. However, there are few experimentally accessible quantities that are closely related to the electrostatic potential. Recent studies by the vibrational Stark effect spectroscopy applied to carbonyl group stretching give some insight into the preorganized electrostatics. The authors calibrated the sensitivity of these vibrations to electric fields and used this calibration to quantitatively interpret the electric field environment experienced by a probe. The approach was pioneered by Boxer's group and applied to quantify the contribution of electric fields to the catalytic rate enhancement by the enzyme ketosteroid isomerase (Sigala et al., 2007; Fried et al., 2014). Much more practical, established and abundant approach is the measurement and calculation of the corresponding p*K*_a_ values.

The p*K*_a_ values of ionizable residues in the enzyme active site are an excellent probe for the electrostatic environment, since p*K*_a_ is very sensitive to any structural change and the associated alteration in the electrostatic potential. Since MAO A and MAO B isozymes are 70% homologous and the overall geometric matching is high (RMSD between the X–ray structures is only 0.66 Å), it is reasonable to expect that their electrostatic potential pattern in the active site will be quite similar and accompanied by a similarity in the p*K*_a_ values of the corresponding residues. It is worth to stress that, in contrast to the point-wise electrostatic potential comparison, p*K*_a_ values can also be determined experimentally. Change of the p*K*_a_ value is also a measure of hydrophobicity: hydrophobic environment does not favor presence of charged species giving rise to significant shifts in the p*K*_a_ values. Despite the fact that monoamines are predominantly monocations at a physiological pH of 7.4, a hydrophobic active site in MAOs has been proposed to favor unprotonated substrates. Furthermore, every catalytic proposal to date has agreed that the substrate must be neutral for the reaction to take place. Active site p*K*_a_ values are difficult to determine experimentally and, similarly, while experimental pH rate profiles can provide tremendous insight, it can be hard to conclusively determine the identity of residues whose protonation state is being affected. For MAO some attempts were performed to determine p*K*_a_ values of some surface residues (Dunn et al., 2008; Repić et al., 2014a). The work of Scrutton and co-workers addressed the p*K*_a_ values of the enzyme active site and the substrates (Dunn et al., 2008). The authors studied pH dependence of MAO A kinetic parameters by stopped-flow and steady state methodology and H/D isotope substitution. The authors came to a conclusion that substrates such as benzylamine, kynuramine, and phenylethyl amine bind in the protonated form, while deprotonation is required for chemical step. Moreover, the authors have demonstrated that the pH dependence of the kinetic isotope effect decreases from ~13 to 8 with increasing pH, leading to the assignment of this catalytically important deprotonation of the amine substrate. The strong H/D kinetic isotope effect dependence gives evidence that at low pH values the substrate deprotonation interferes with the rate-limiting step. Moreover, Scrutton and co-workers suggested that the p*K*_a_ values of the studies substrates are decreased for about 2 p*K*_a_ units when transferring the substrate from aqueous solution to the enzyme. To get an insight into the nature of the MAO active sites, the p*K*_a_ values of a few ionisable residues were calculated in order to compare the electrostatic potential pattern in the active sites of both isoforms (Dunn et al., 2008). Well-converged free energy calculations of the p*K*_a_ values were performed using the MOLARIS program package together with an all-atom representation of the solvated enzymes. In line with the general consensus that dopamine enters the chemical step in a neutral form, a neutral dopamine molecule was manually docked in the active site in a way suitable for the catalytic step. We chose only one substrate on purpose in order to avoid the effects associated with ligands of different sizes on the p*K*_a_ values. p*K*_a_ calculations were performed using the semimacroscopic protein dipole/Langevin dipole approach of Warshel and co-workers in its linear response approximation version (PDLD/S–LRA), allowing for converged free energy calculations (Lee et al., 1993; Schutz and Warshel, 2001).

**Figure 1:**
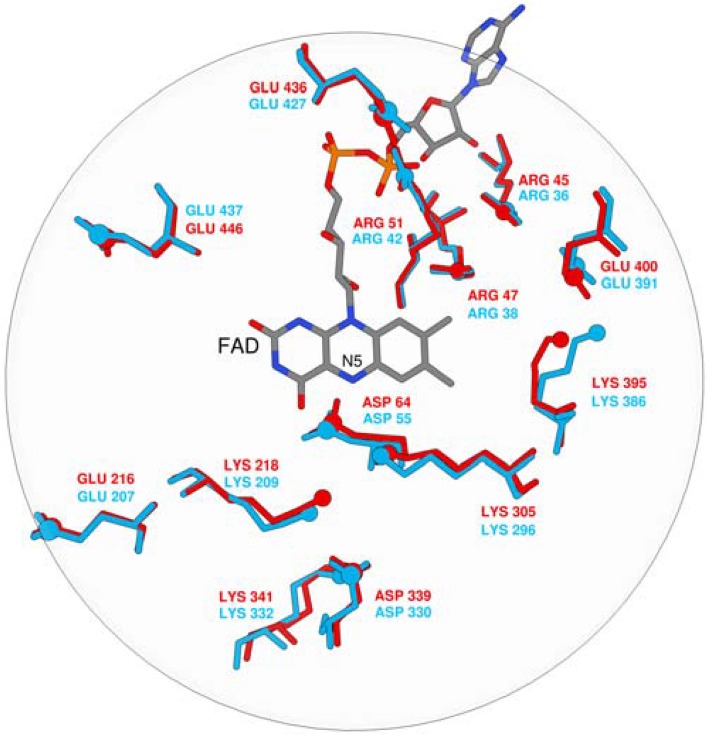
**Superposition of FAD and ionisable residues surrounding the active site (MAO A is red, MAO B is blue)**.

The calculated p*K*_a_ values are sensitive to the applied external dielectric constant during simulations. The choice of the correct dielectric constant to describe the protein interior is a very complex issue and has been the subject of heated debates over the years (Schutz and Warshel, 2001). In our work, we employed ε = 10–16, still, due to the focus on the relative difference between p*K*_a_ values in MAO A and MAO B, the choice would not change the qualitative picture and is thus of lesser importance. Specifically, we have examined the p*K*_a_ values of four tyrosine residues that are part of the so-called aromatic cage and a Lys residue close to the reacting atoms of FAD. Among isoforms, the average absolute differences over a dielectric constant of 10–16 in the corresponding p*K*_a_ values are assumed to be 0.05, 0.07, 0.12, 0.75, and 1.23 for MAO A/B pairs Tyr69/Tyr60, Lys305/Lys296, Tyr407/Tyr398, Tyr444/Tyr435, and Tyr197/Tyr188, respectively. These results clearly demonstrate that, for both isozymes, the p*K*_a_ values for the Tyr and Lys residues in question are not substantially different (1.23 p*K*_a_ units at maximum), thus providing strong evidence that the electrostatic potential pattern in the active sites of both isozymes is very closely matched. Since enzymes work by preorganized electrostatics, the same electrostatic environment cannot be at the same time suitable for optimal solvation of the transition state with a positive and a negative charge build-up, as would be the case in the hydride and polar nucleophilic mechanisms. Superimposition of both experimental X-ray structures also reveals a high similarity in the spatial configuration of charged groups in MAOs (Figure 1). It is therefore unlikely that MAO A and MAO B would work by different chemical mechanisms on the same family of substrates, thus contradicting an interesting proposal of Orru and co-workers (Orru et al., 2013). The p*K*_a_ value of the bound dopamine (8.8) is practically unchanged compared to the corresponding value in aqueous solution (8.9), as would be expected from a charged amine placed in a hydrophobic active site consisting of aromatic moieties (Li et al., 2006). The MAO active sites are in this respect not hydrophobic and practically unchanged dopamine p*K*_a_ relative to aqueous solution may be attributed to favorable cation–π interactions between the dopamine –${\text{NH}}_{3}^{+}$ group and aromatic moieties, which provide a stabilizing effect to the charged fragment. Our results can be contrasted with the study of Scrutton and co-workers reporting the p*K*_a_ shift of the substrates for about 2 p*K*_a_ units. The discrepancy can be, among other factors, explained by the sizes of different substrates.

## Mechanistic studies of monoamine oxidase by quantum mechanical cluster model

The general reaction of flavin amine oxidases, including MAO, can be divided into two half-reactions. In the reductive half-reaction, a hydride equivalent is transferred from the substrate to the flavin, thus reducing it to FADH_2_, while the oxidative half-reaction involves the oxidation of the reduced flavin back to FAD by molecular oxygen, producing H_2_O_2_ (Scheme 1).

The chemical mechanism of the reductive half-reaction in MAO has been the source of controversy and debate (MacMillar et al., 2011). Oxidation of an amine substrate necessarily involves the removal of two protons and two electrons as the carbon–nitrogen single bond is converted to a double bond. Three-dimensional structures of MAO A and MAO B isoforms (Binda et al., 2002; De Colibus et al., 2005; Son et al., 2008), together with extensive kinetic and spectroscopic studies on mutant enzymes (Binda et al., 2002; De Colibus et al., 2005; Li et al., 2006), have led researchers to propose three possible catalytic scenarios for MAO (MacMillar et al., 2011): (a) the direct hydride mechanism, (b) the radical mechanism, and (c) the polar nucleophilic mechanism. Studies on deuterated substrate analogs have suggested that the rate-limiting step is the cleavage of a carbon-hydrogen bond vicinal to the amino group (Klinman and Matthews, 1985) and, hence, the catalytic proposals differ in the nature of the hydrogen being transferred, namely a hydride (H^−^) in (a), a hydrogen atom (H^•^) in (b), and a proton (H^+^) in (c) (Walker and Edmondson, 1994; Wang and Edmondson, 2011), commonly by the flavin N5 atom. In contrast, the other hydrogen from the amino N–H moiety is generally proposed to be abstracted as a proton (Harris et al., 2001). Thus, establishing the MAO catalytic mechanism necessarily requires the knowledge of the timing of the removal of hydrogens from both the carbon and nitrogen atoms.

The possibility of the hydride mechanism for MAOs was based on their structural similarities to flavoprotein D-amino acid oxidases (DAAO), for which both kinetic measurements (Kurtz et al., 2000; Ralph et al., 2007) and related calculations (Ralph et al., 2007) suggested a hydride or a single electron transfer. Additionally, deuterium and ^15^N kinetic isotope effect studies of flavin amine oxidases including DAAO (Kurtz et al., 2000), tryptophan-2-monooxygenase (TMO; Ralph et al., 2006), and *N*-methyltryptophan oxidase (MTOX; Ralph et al., 2007), are most consistent with a hydride transfer mechanism. However, Erdem et al. (2006) assumed that a hydride mechanism was unlikely, and concluded that in MAO it would be associated with a barrier too high to be readily crossed. In addition, a combined ^15^N and deuterium isotope effects demonstrated that the C–H bond cleavage is not concerted with the rehybridization of the substrate amino group (MacMillar et al., 2011), which seem to rule out the feasibility of a concerted hydride transfer mechanism.

According to Silverman and co-workers (Silverman, 1995) the radical mechanism is initiated by a single-electron transfer from the substrate to the flavin, producing an aminium radical cation and a flavin semiquinone as transient intermediates. The lowered p*K*_a_ of the α–CH bond of the aminium radical cation can result in either a stepwise (Silverman, 1995) or concerted (Vintém et al., 2005) deprotonation and a second electron transfer producing reduced flavin and the iminium ion. The main supporting evidence for the radical mechanism is the observation that both MAOs are inactivated by cyclopropylamine analogs with subsequent ring opening, a process characteristic of radical reactions (Silverman, 1983). An argument against the radical mechanism has been the failure of any laboratory to detect semiquinone radical species during turnover in a stopped-flow monitored reduction or using radical traps (see later). The chemistry of the cyclopropylamine probes and resulting products supported the view that single electron transfer is possible at least with these compounds, but the lack of inactivation by, and a ring-opened product from trans-2-phenyl(aminomethyl)cyclopropane suggested that other mechanisms must be possible (Fitzpatrick, 2010). In a modified single electron transfer mechanism, the involvement of protein-based radicals has also been proposed but questioned (Rigby et al., 2005; Kay et al., 2007). Mutation of the substrate-orienting tyrosines and isotopic labeling of the tyrosines in MAO A, and the fungal counterpart MAO B, provide evidence for radicals delocalized away from the active site (Dunn et al., 2010).

The experiments by Edmondson and co-workers (Walker and Edmondson, 1994), as well as related electron paramagnetic resonance studies (Tan et al., 1983; Miller et al., 1995; Nandigama and Edmondson, 2000) and stopped-flow kinetic determinations (Nandigama and Edmondson, 2000), failed to provide any evidence for radical intermediates, and no influence of the magnetic field on the kinetics of enzyme reduction was observed (Miller et al., 1995). In addition, Taft correlation studies of Miller and Edmondson with benzylamines showed that attaching the electron-withdrawing groups to the substrate *para*-position increases the rate of the reaction in both human (Miller and Edmondson, 1999a) and rat (Wang and Edmondson, 2011) MAO A, implying negative charge build-up on the substrate α–carbon atom, thus suggesting that proton transfer is an integral part of the rate limiting step. This led authors to propose the polar nucleophilic mechanism for MAO A (Miller and Edmondson, 1999b), originally formulated by Hamilton (Hamilton, 1971). This mechanism involves the creation of a highly energetic substrate–flavin adduct which then decomposes to the protonated imine, with proton abstraction concerted with either the adduct formation or the product formation. The crucial issue related to this mechanism is what moiety on the enzyme would be strong enough base to perform this task, since the p*K*_a_ of a benzyl proton is expected to be around 25 (Smith and March, 2001). Structural analysis of both MAO isoforms showed there are no active site basic residues that could act as proton acceptors. Also, no direct evidence for a stable amine–flavin adduct has been found experimentally. A study on human MAO B, however, showed an inverse Taft correlation (Orru et al., 2013), supporting the hydride transfer mechanism, which led the authors to propose different mechanisms for two isozymes: H^+^ transfer in MAO A and H^−^ transfer in MAO B, although, the same authors had previously ruled out H^−^ transfer in human MAO B, based on the nitrogen secondary kinetic isotope effects (MacMillar et al., 2011). Based on the similarities in the continuous wave electronic paramagnetic resonance spectrum between MAO and *D*-amino acid oxidase, determined to operate by the H^−^ transfer mechanism (Umhau et al., 2000), Kay and co-workers suggested a hydride transfer mechanism should be re-examined for MAO (Kay et al., 2007). Therefore, it is clear that, despite the widespread use of MAO inhibitors, the mechanism of MAO catalysis is not unambiguously determined which calls for its rationalization using advanced computational techniques.

**Figure 2:**
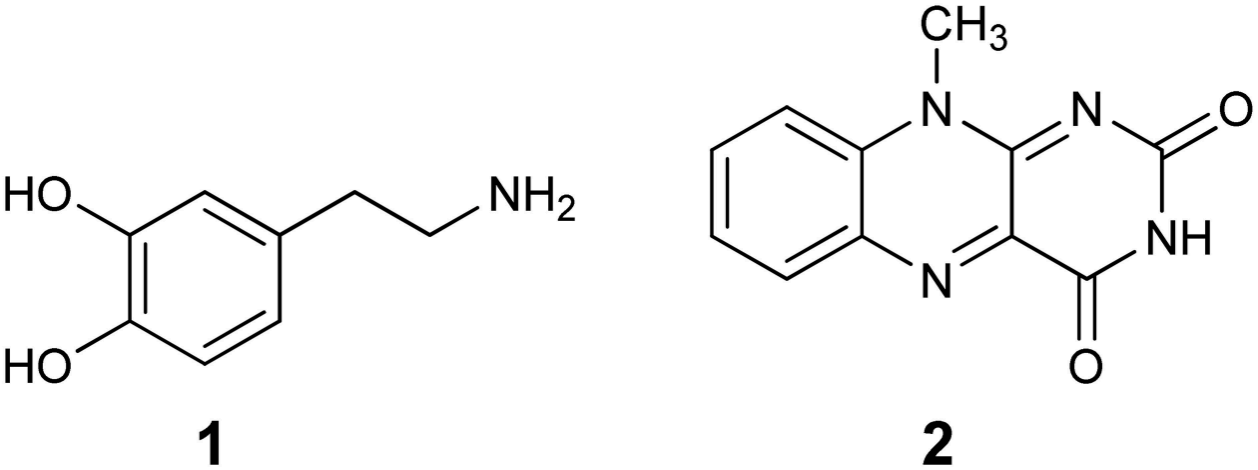
**Structures of dopamine (1) and isoalloxazine moiety (2) of the FAD cofactor used in the cluster model of the MAO active site**.

**Figure 3:**
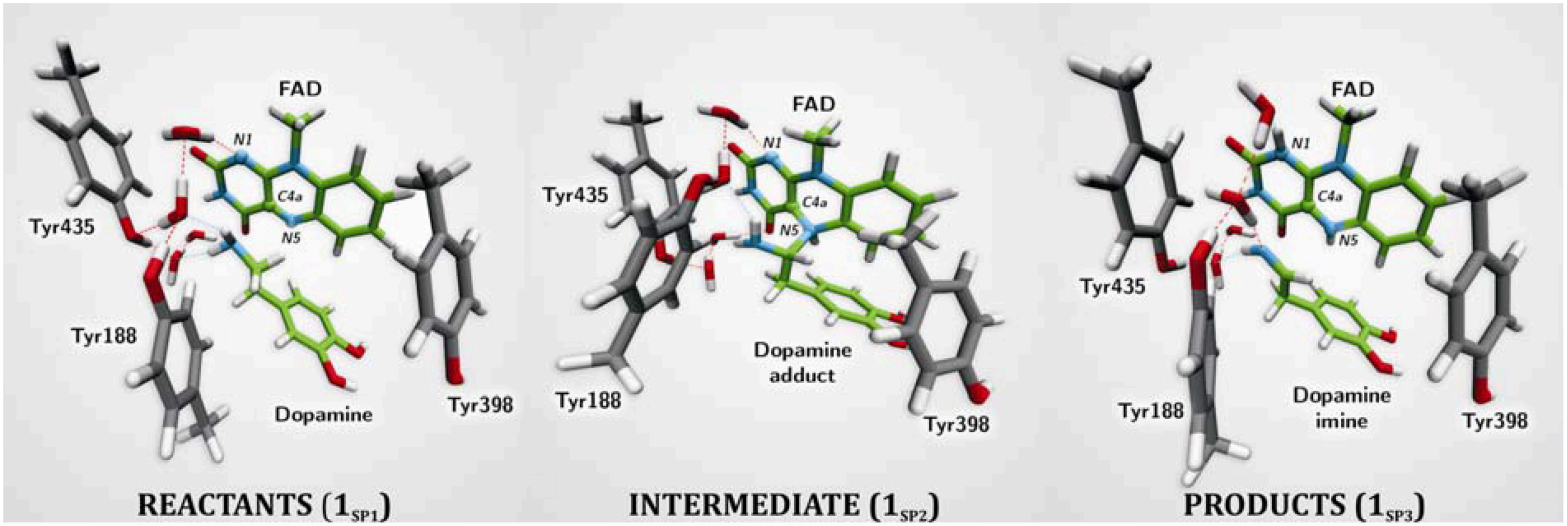
**Structures of relevant stationary points for the newly proposed two-step MAO catalytic hydride mechanism for the degradation of dopamine 1**.

The first computational study on the complete mechanism of MAO catalysis was performed by our groups (Vianello et al., 2012), employing DFT methodology within the cluster model of the enzyme. The starting point for our calculations was the high-resolution (1.6 Å) X-ray structure of MAO B complexed with 2-(2-benzofuranyl)-2-imidazoline (accession code 2XFN; De Colibus et al., 2005). We truncated the enzyme to the flavin moiety (isoalloxazine group **2**, Figure 2) and three tyrosine side-chains (*p*-hydroxytoluenes of Tyr188, Tyr398, and Tyr435), which all form the mentioned hydrophobic “aromatic cage” (Li et al., 2006; Akyuz et al., 2007). Previously, we calculated the p*K*_a_ values of the Tyr residues with bound dopamine (Borštnar et al., 2012), and obtained an upward shift to 13.0–14.7 (p*K*_a_ for Tyr in aqueous solution is 10.1). This clearly confirmed the hydrophobic nature of the active site, which indicates that gas-phase calculations on truncated MAOs are reliable. Also, we showed that the p*K*_a_ value of bound dopamine changes to only 8.8 (8.9 in aqueous solution), a result of stabilizing cation–π interactions with the tyrosine side-chains (Borštnar et al., 2012). This implies that dopamine binds to the MAO active site as a protonated monocation, but the free-energy cost to deprotonate it to the bulk, being as low as 1.9 kcal mol^−1^, allows it to enter the chemical step either as an ionized or neutral molecule, which led us to consider both alternatives. Based on crystal structures (Binda et al., 2002; De Colibus et al., 2005; Son et al., 2008) and our MD simulations (Borštnar et al., 2012) that both indicate the presence of few water molecules in the active site, our model also included four crystal water molecules (HOH_2157_, HOH_2181_, HOH_2329_, and HOH_2372_). It turned out that two of those are chemically involved in catalysis. We manually placed dopamine **1** within the cluster, resulting in the initial stationary-point (**SP**) complexes (Figure 3). The system was modeled at the (CPCM)/M06–2X/6–311++G(2df,2pd)//(CPCM)/M06–2X/6–31+G(d) level of theory employing the M06–2X functional designed by Zhao and Truhlar to accurately reproduce thermodynamic and kinetic parameters (Zhao and Truhlar, 2008, 2011; Bell and Head-Gordon, 2011; Cheong et al., 2011; Picek et al., 2015; Saftić et al., 2015), being particularly successful in treating non-bonding interactions. To account for the polarization effects caused by the rest of the enzyme, we included a conductor-like polarizable continuum model (CPCM) (Cossi et al., 2003) with a dielectric constant of ε = 4, taking the rest of the parameters for pure water, as employed in many studies by Siegbahn, Himo and their co-workers in elucidating the catalytic mechanism of a large variety of enzymes (Himo, 2006; Siegbahn and Borowski, 2006; Siegbahn and Himo, 2011).

For **1_SP1_** (Figure 3) we first considered the single-electron radical mechanism, which would initially create a biradical either in the singlet or triplet electronic states. (CPCM)/UM06–2X/6–31+G(d) calculations for the triplet state produced the system 54.2 kcal mol^−1^ higher in energy than **1_SP1_**. For the singlet state, we employed “symmetry-broken” open-shell calculations, defining dopamine and the rest of the system as two different fragments each having an unpaired electron of opposite spin, which resulted in a stable wave-function with both the energy and electron distribution identical to that in **1_SP1_**. Overall, this suggests that the singlet-state biradical is non-existent, whereas the free-energy cost to generate the triplet is too high for an efficient catalysis. This agrees well with the experimentally observed mismatch between the oxidation/reduction potentials of the FAD cofactor, which is too low (−0.2 V; Newton-Vinson and Edmondson, 1999) for it to be an effective oxidant of the neutral amine (around +1.0–1.5 V) (Hull et al., 1969). On top of the fact that there is no experimental evidence for a radical intermediate, our results suggest that it is very unlikely that a radical pathway is feasible and we did not consider it further.

The polar nucleophilic mechanism is another alternative for the amine oxidation and involves proton abstraction from the α–carbon atom as the rate-limiting step (Miller and Edmondson, 1999b). The crucial issue relating to this mechanism is what moiety on the enzyme would be a strong enough base to perform this task, because the p*K*_a_ of a benzyl proton is expected to be around 25 (Smith and March, 2001). Structural analysis of both MAO isoforms shows there are no basic active-site residues that could act as proton acceptors (Binda et al., 2003). Edmondson and coworkers upheld their arguments by stating that in MAOs the flavin is bent by around 30° from planarity about the N5–N10 axis (Binda et al., 2003), which enhances the basicity of the N5 atom and depletes the electron density on the C4a atom, thus facilitating substrate–flavin complex formation with the former flavin site making subsequent proton abstraction possible. We feel that, even if all of these effects are operational, it would still be insufficient to downshift the substrate p*K*_a_ value by around 10–15 units in order to make the α–CH bond acidic enough for an efficient catalysis. Furthermore, a relaxed-geometry scan of the latter bond, by compressing it with 0.1 Å increments, showed no indication of the formation of a stable complex. In addition, NBO charges on the flavin C4a and N5 sites and the substrate N atom in **1_SP1_** are 0.13, −0.35, and −0.97|e|, respectively, which demonstrate that they do not change much from the values found in isolated flavin **2** and dopamine **1** (0.10, −0.34, and −0.93|e|; Table 1), and that there is no charge transfer already in the Michaellis complex, as proposed by some authors in favor of the polar nucleophilic mechanism (Abad et al., 2013). This all indicates that neutral amines do not exhibit the necessary nucleophilicity to readily add to the flavin C4a position, in agreement with the fact that no direct evidence for a stable amine–flavin adduct has been found experimentally. Taken all together, these results also led us to rule out this mechanism as feasible.

**Table 1:** **Evolution of atomic charges during the C(α)–H hydride abstraction reaction from dopamine to flavin as obtained with the NBO approach at the (CPCM)/M06–2X/6–31G(d) level of theory**.

**System**	**Atom/group**	**Isolated**	**Reactants (1_SP1_)**	**TS (2_TS1_)**	**Intermediate (2_SP2_)**
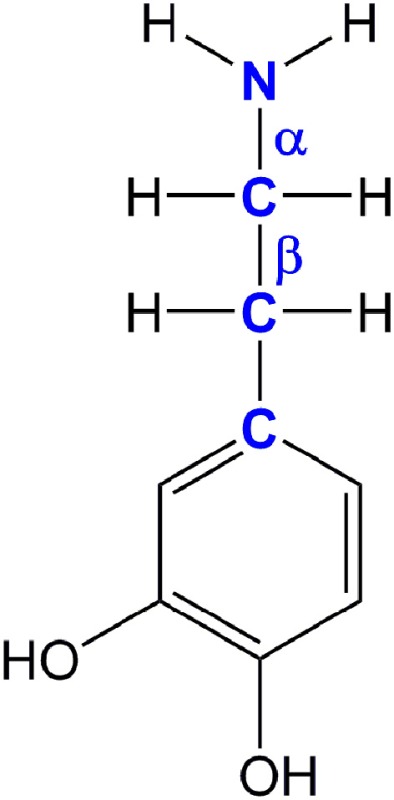	N(amino)	−0.93	−0.97	−0.79	−0.90
	α–C	−0.26	−0.25	−0.11	0.16
	β–C	−0.50	−0.49	−0.50	−0.52
	C1(phenyl)	−0.06	−0.04	−0.06	−0.07
	dopamine	0.00	−0.03	0.31	0.43
	N5(flavin)	−0.34	−0.35	−0.50	−0.50
	N1(flavin)	−0.63	−0.68	−0.71	−0.70
	flavin	0.00	0.01	−0.29	−0.35

The geometry of stationary structure **1_SP1_** (Figure 3) suggests that the following two pathways are possible. The substrate amino group is connected through two active-site water molecules to the flavin N1 atom, which is the most basic position within the isoalloxazine moiety (Vianello et al., 2012). This implies that the substrate could be first activated by amino deprotonation to the flavin N1 site. Also, the α–C(substrate) · · ·N5 bond length in **1_SP1_** is 3.198 Å, being sufficiently short to suggest that the substrate is properly oriented for a direct α–CH abstraction.

**Figure 4:**
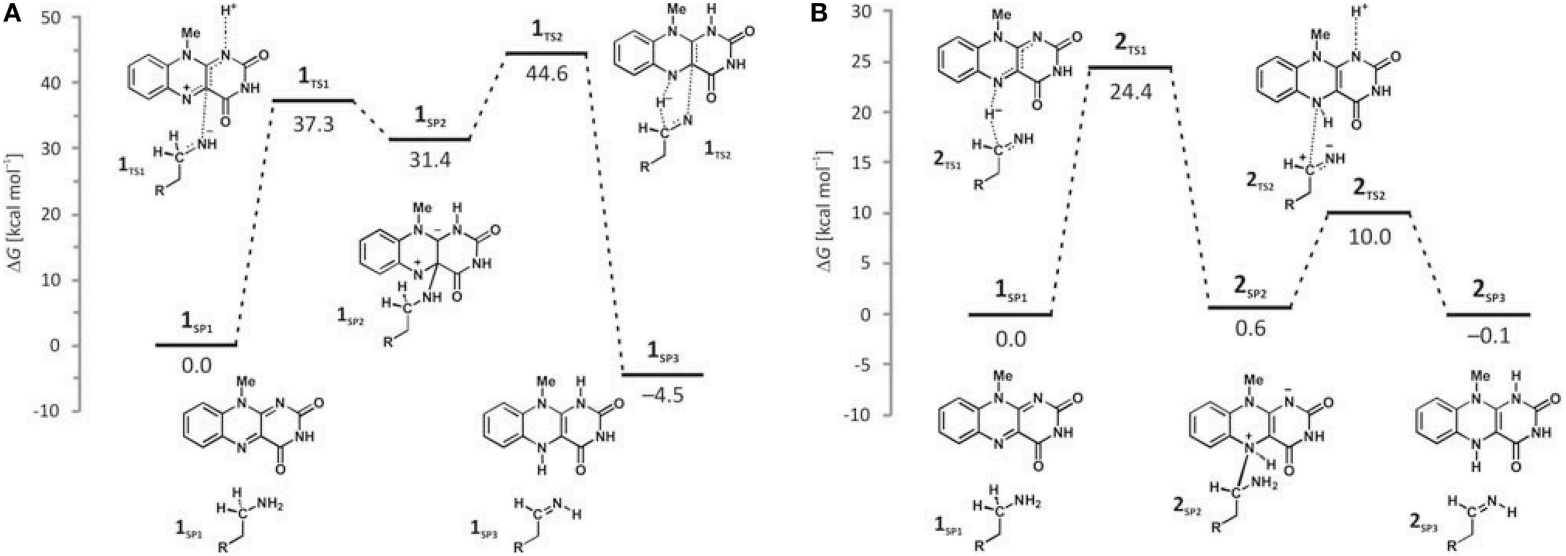
**Free-energy profiles for the MAO catalyzed amine degradation reaction initiated by either amino deprotonation (A), or a direct hydride abstraction (B) as obtained with the (CPCM)/M06–2X/6–311++G(2df,2pd)//(CPCM)/M06–2X/6–31+G(d) model employing the cluster model of the enzyme**.

Deprotonation of the neutral substrate amino group is feasible, but the process is associated with a large barrier of 37.3 kcal mol^−1^ (Figure 4; Vianello et al., 2012). The transition-state structure **1_TS1_** has one imaginary frequency of 270*i* cm^−1^ and the corresponding eigenvector represents a proton transfer to the flavin N1 atom assisted by two water molecules by the de Grotthuss mechanism (de Grotthuss, 1806). Such a high energy requirement is rationalized by the large difference in the p*K*_a_ values between proton donor and acceptor sites. For the flavin N1–H deprotonation p*K*_a_ was measured to be around 7.0 (Macheroux et al., 2005), whereas amine deprotonation typically has a p*K*_a_ of around 35 (Smith and March, 2001). Upon proton removal, the anionic substrate turns nucleophilic and covalently binds to the flavin C4a atom. The formed complex facilitates subsequent H^−^ abstraction from the α–CH group, requiring only 13.2 kcal mol^−1^ to arrive at the transition-state structure **1_TS2_** (υ_imag_ = 871*i* cm^−1^). Abstraction of the H^−^ is concerted with the loosening of the N(substrate) · · ·C4a bond from 2.439 Å in **2_TS2–N_** to 3.505 Å in the final products **1**_SP3_, being reduced flavin FADH_2_ and neutral imine. This pathway is associated with collective activation energy of 44.6 kcal mol^−1^, which is too high to be feasible. In an analogous pathway, only starting from the substrate with the protonated amino group, the flavin N1 deprotonation of the –${\text{NH}}_{3}^{+}$ group becomes easier requiring only 20.7 kcal mol^−1^ (υ_imag_ = 980*i* cm^−1^) The subsequent α–hydride abstraction by the flavin N5 atom costs an additional 24.4 kcal mol^−1^ to add up to an overall reaction free-energy barrier of 43.1 kcal mol^−1^, yielding protonated imine, R–CH_2_CH_2_ = ${\text{NH}}_{2}^{+}$. Although the collective activation barrier for this process is slightly lower than with the unionized substrate, it is still too high for this mechanism to be plausible.

Traditional notion of the hydride abstraction should generate positive charge on the substrate α–carbon atom, which would seem to contrast the aforementioned positive Taft correlation (Miller and Edmondson, 1999a) that suggested negative charge development on the stated carbon atom. However, due to H^−^ abstraction, the depletion of electron density on the α–carbon atom is completely outperformed by the strong electron-donating ability of the vicinal amino group, as evidenced by a decrease in both the corresponding N(amino)–C(α) bond length and the nitrogen atomic charge from 1.466 Å and 0.97|e| in **1_SP1_** to 1.354 Å and −0.79|e| in **2_TS1_**. As a result, the charge on the α–carbon atom changes from −0.25|e| in **1_SP1_** to −0.11|e| in **2_TS1_**, surprisingly preserving a significant portion of the negative charge (Table 1). More importantly, the charge on the dopamine β–carbon atom, which is in terms of *para*-substituent effects analogous to the benzylamine α–carbon atom, even increases from −0.49|e| (**1_SP1_**) to −0.50|e| (**2_TS1_**), which, taken all together, gives strong evidence to why this reaction is facilitated by the electron-withdrawing *para*-substituents on the aromatic ring, thus putting our results in firm agreement with the work of Miller and Edmondson (1999a,b). After the flavin accommodates H^−^, its N5 atom becomes sp^3^–hybridized with excess negative charge, having enough nucleophilicity to form a covalent bond with the thus formed cationic substrate (Figures 3, 4). Macheroux et al. (2005) measured the p*K*_a_ value of the fully hydrogenated flavin N(5)–H_2_ moiety to be p*K*_a_ = 4, which indicates that the N(5)–H^−^ group possesses sufficient basicity to act as a base. The calculated substrate–flavin interaction free energy in **2**_SP2_ is as high as 27.7 kcal mol^−1^, which, together with the corresponding N5–C(α) bond length of only 1.703 Å, suggests that the formed complex is rather strong. With the complex formation, the necessity for another step in this process becomes apparent, which represents an important advantage over other mechanistic proposals that all advise protonated imine as the final product. We disagree with the latter for two reasons. First, it would be difficult for a protonated imine to leave the active site, because on its way out it would relatively strongly bind to the “aromatic cage” through favorable cation–π interactions (Borštnar et al., 2012). Secondly, it is well-established that the final imine hydrolysis to aldehydes occurs non-enzymatically outside the MAO (Edmondson et al., 1993; Woo and Silverman, 1995). However, the protonated product would immediately be hydrolyzed by the nearest water molecule within the enzyme, because in organic chemistry this reaction readily proceeds with the protonated imine under acidic conditions (Smith and March, 2001).

The next step involves amino group deprotonation by the flavin N1 atom with an activation free energy of 9.4 kcal mol^−1^ (Figure 4), being concerted with the weakening of the adduct N5(flavin) · · ·Cα(dopamine) bond. The transition-state **2_TS2_** (υ_imag_ = 933*i* cm^−1^) again describes a de Grotthuss-type proton transfer proceeding with the two active-site water molecules. Upon deprotonation, the system is stabilized by 10.1 kcal mol^−1^ to **2**_*SP*3_, making the whole reaction energetically feasible and yielding the neutral trans-imine and the fully reduced flavin (FADH_2_) as final products (Figures 3, 4). It has to be strongly emphasized that the presence of the acidic N–H bond enables the completion of MAO turnover and explains why many alkyl- and arylamines change from being MAO substrates to MAO inhibitors upon *N*,*N*-dimethylation (Ding et al., 1993). The fact that dopamine is converted into a neutral imine is significant, because this suggests it will predominantly remain unprotonated in the hydrophobic active site, based on consideration of the p*K*_a_ values of similar unconjugated imines, which are, as a rule, found to be below the physiological value of 7.4 (for example, the p*K*_a_ value of Me_2_C=N−Me is 5.5) (Smith and March, 2001), ensuring that the neutral product could go past the “aromatic cage” on its release from the active site. This, however, does not rule out the possibility that, when it is finally liberated from the enzyme, the imine formed could be readily protonated, because, during its departure, changes in the environment could make the protonation feasible. It is also possible that the protonation occurs already close to the active site since hydrated enzyme represents proton rich environment. This would then fully agree with Edmondson et al. (1993), who showed that the protonated *p*-(dimethylamino) benzylimine is released from the enzyme. Also, the fact that flavin is fully reduced to FADH_2_ enables an important prerequisite for MAO regeneration by molecular oxygen to revert flavin into its oxidized form (FAD) by creating hydrogen peroxide (H_2_O_2_), a reaction for which two hydrogen atoms are required (Scheme 1). Please note that hydrogen peroxide further reacts and produces reactive oxygen species that are responsible for the oxidative stress and neurodegeneration (*vide infra*).

**Figure 5:**
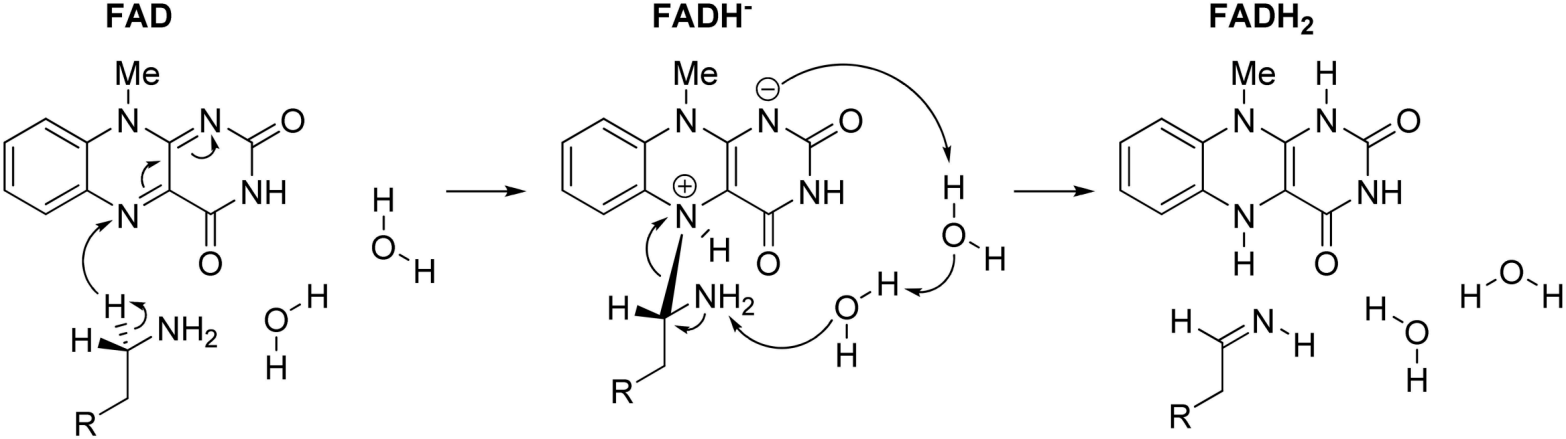
**Complete two-step mechanism for MAO catalyzed amine degradation**. The first step involves H^−^ abstraction from the substrate to form the flavin–substrate adduct, which then decomposes to the final products, namely neutral imine and fully reduced flavin, FADH_2_, a reaction promoted by amine deprotonation facilitated by two water molecules.

In concluding this section, let us emphasize that presented results have convincingly demonstrated the prevailing feasibility of the two-step hydride transfer mechanism (Figure 5). In recent years, there have been several additional computational studies showing its prevailing feasibility in MAO (Akyüz and Erdem, 2013; Atalay and Erdem, 2013; Zapata-Torres et al., 2015), or some other flavoenzymes (Kopacz et al., 2014; Karasulu and Thiel, 2015). Furthermore, in what follows, we will demonstrate that when we moved from the QM-only cluster model toward including the full enzyme structure, *via* the Empirical Valence Bond QM/MM approach, the calculated activation free energy for the MAO B catalyzed degradation of dopamine drops down to ΔG^‡^ = 16.1 kcal/mol (Repić et al., 2014b), being in excellent agreement with the available experimental value of 16.5 kcal/mol (Walker and Edmondson, 1994; Wang and Edmondson, 2011), thus providing a strong support for our mechanistic picture.

## Multiscale simulation of MAO catalyzed chemical step for different substrates

In this section, we focus to the extension of the static cluster model study of the rate-limiting step in MAO B to a full enzyme dimensionality using multiscale EVB approach with extensive thermal averaging allowing for a direct evaluation of the enzyme catalytic effect compared to the reference reaction in the gas phase or aqueous solution (Warshel, 2014).

For enzyme reactions and reactions in polar solutions, transition state theory is practically always valid. To calculate the rate constant for the reaction it is, therefore, necessary to know the barrier height in terms of free energy. Faster reaction has lower barrier than the slower one. Catalysis is the reaction rate enhancement relative to the reference reaction and catalyzed reaction has, therefore, lower free energy of activation than the uncatalyzed reaction. The natural reference state for an enzymatic reaction is the corresponding uncatalyzed reaction in aqueous solution, since biology occurs in this medium. However, considering the lack of direct experimental kinetic data in aqueous solution, our calculated reference state for the MAO B catalyzed reaction is the corresponding hydride transfer in the gas phase, as this is the state for which one is most likely to obtain reliable estimates of the activation barrier using quantum chemical approaches. For the gas phase reference reaction between lumiflavin **2** and dopamine **1** we used the optimized geometries of the reactants and transition state from the cluster study. The gas phase energetics of the reaction were calculated using the M06–2X density functional in conjunction with the 6–31+G(d,p) basis set as described in previous section. For simulation using full dimensionality of the enzyme with included water molecules, atomic coordinates were obtained from the high-resolution (1.6 Å) crystal structure of MAO B obtained from the Protein Data Bank (accession code 2XFN). The charges of all relevant structures were fitted to the electrostatic potential and calculated with inclusion of the solvent reaction field at the (CPCM)/B3LYP/6–311G(d,p) level. The dopamine substrate was manually docked into the MAO B active site. EVB calculations were performed *in vacuo* representing the reference state, aqueous solution as well as full dimensionality of the MAO B enzyme, to examine the effect of different environments on the reaction barrier. For all EVB calculations the same EVB region was used, consisting of lumiflavin moiety and dopamine for a total simulation time of 15.3 ns, while for other technical details the reader is referred to the original publication (Repić et al., 2014b). All EVB calculations were performed using the standard EVB free energy perturbation/umbrella sampling (EVB–FEP/US) procedure (Kamerlin and Warshel, 2011b) and the MOLARIS simulation package in combination with the ENZYMIX force field (Schutz and Warshel, 2001).

In order to study enzyme catalysis, it is highly desirable to have the experimental kinetic data for the reference reaction in aqueous solution. To our best knowledge experimental kinetic data for the reaction between flavin and dopamine in aqueous solution are not available. There are also no available data about the kinetics of uncatalyzed reactions between flavins and phenethylamines or some suitable model compounds. Some studies has shown that flavins react with amines and alcohols in aqueous solution on a timescale of days, but no further kinetic data were provided and, therefore, experimental value for activation free energy of the reference reaction cannot be deduced (Brown and Hamilton, 1970; Kim et al., 1993). Therefore, we parameterized our EVB Hamiltonian to reproduce reliable M06–2X/6–31+G(d,p) energetics *in vacuo* (Δ$G_{\text{gas}}^{‡}$ and Δ$G_{\text{gas}}^{0}$). The system was subsequently moved to aqueous solution and to the MAO B active site using the same parameter set (EVB off-diagonal term plus the gas phase shift). This is a valid approximation, due to the demonstrated phase-independence of the EVB off-diagonal (H_*ij*_) coupling term (Hong et al., 2006).

**Figure 6:**
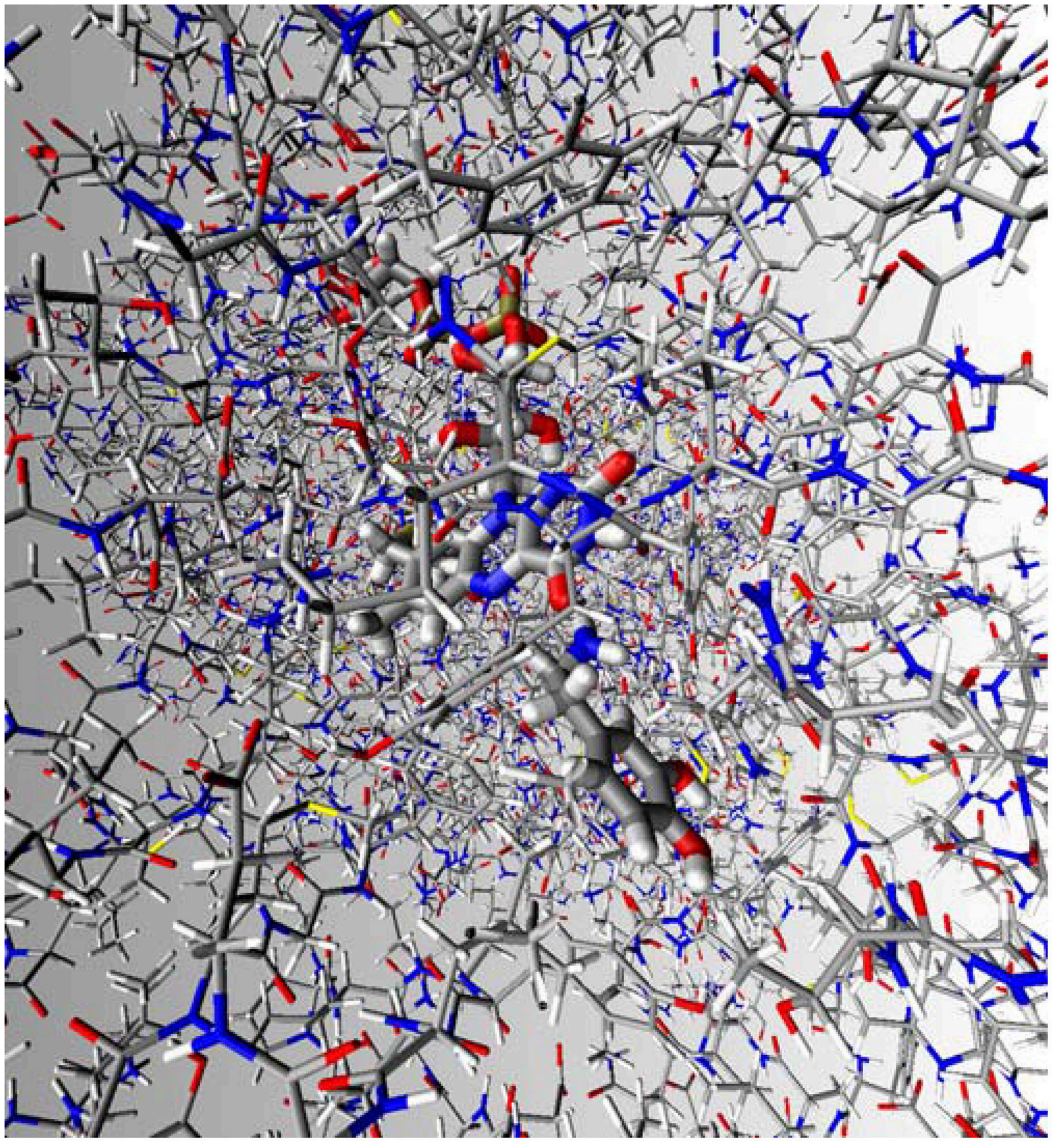
**Structure of the MAO B active site with the reactive neutral dopamine**. The FAD prosthetic group is shown in orange, dopamine in light blue, and Lys296 in violet.

To model the hydride transfer step using EVB, the activation free energy (Δ${\text{G}}_{\text{gas}}^{‡}$) and the free energy (Δ${\text{G}}_{\text{gas}}^{0}$) of the reference reaction need to be known. While it is straightforward to obtain Δ${\text{G}}_{\text{gas}}^{‡}$, the calculation of Δ${\text{G}}_{\text{gas}}^{0}$ is complicated by the fact that, in the gas phase, the resulting FADH_2_ anion and dopamine cation form a bound adduct immediately upon hydride transfer. In order to circumvent this issue we selected the point in the Intrinsic Reaction Coordinate (IRC) profile where the structure of FADH^−^ moiety matches the geometry of the gas-phase optimized FADH^−^. In addition, the IRC profile shows a shoulder in the potential energy surface around the same point, thus suggesting a transient intermediate. Full geometrical optimization of the reactant, the transition state, and the adduct, including the thermal correction to Gibbs free energy, shows that the transition state and adduct are 32.8 and 10.0 kcal mol^−1^ higher in energy than the reactants, respectively. Taking into account the thermal correction, the product is 24.9 kcal mol^−1^ higher in energy than the reactants. The EVB gas-phase shift and coupling parameter were thus parameterized to reproduce these Δ${\text{G}}_{\text{gas}}^{‡}$ and Δ${\text{G}}_{\text{gas}}^{0}$ values. Using the same parameters as in the gas-phase EVB simulation the reaction in aqueous solution and in the enzyme was simulated. In aqueous solution the activation free energy is still high, 26.5 kcal mol^−1^, it is lowered by 6.3 kcal mol^−1^ relative to the gas phase. Even more pronounced is the effect of the aqueous environment to the free energy of reaction, which is lowered to 4.2 kcal mol^−1^. By inclusion of the full dimensionality of the enzyme environment, reduction of the barrier to 14.2 kcal mol^−1^ is observed and the reaction becomes exergonic with a reaction free energy of −0.6 kcal mol^−1^ (Repić et al., 2014b). The reduction in the activation barrier compared to the aqueous solution is 12.3 kcal mol^−1^, which corresponds to a rate-enhancement of more than nine orders of magnitude. The reaction is not excessively exergonic, allowing for the reverse reaction in the case of product overproduction. Experimental studies revealed that the substrates are bound to the MAO B active site in the protonated form, while they enter the reaction only if they are neutral. We calculated the p*K*_a_ value for dopamine placed at the MAO B active site and we have shown that it is not significantly changed relative to its value in water. The reversible work necessary for dopamine deprotonation can be calculated then analytically and its value is 1.9 kcal mol^−1^. When this correction is added to the calculated barrier of 14.2 kcal mol^−1^, it gives an activation free energy of 16.1 kcal mol^−1^. The latter is in excellent agreement with the experimental value of 16.5 kcal mol^−1^ (Edmondson et al., 2009) thus strongly supporting the proposed hydride transfer mechanism. A snapshot from the simulation of MAO B active site with the reactive dopamine molecule is shown in Figure 6.

The Lys296 residue is a part of the FAD–H_2_O–Lys296 motif conserved in many flavin-dependent oxidases and we speculated that it might be important in the MAO catalysis. We have performed our calculations with both the neutral and protonated form of Lys296 to quantify the effect of the ionization state of this residue on the hydride transfer reaction. Our results show that the protonation state of Lys296 does not affect the reductive half-reaction of MAO B and that the barrier for the reaction is virtually unchanged at 14.0 kcal mol^−1^. One may speculate that the protonation state of Lys296 may be relevant for the oxidative half-reaction, what is in accordance with the conclusion for mouse polyamine oxidase (Henderson Pozzi and Fitzpatrick, 2010).

Following the same methodology, we also addressed the catalytic step of MAO A catalyzed decomposition of noradrenaline (Poberžnik et al., unpublished data). We showed that MAO A lowers the activation barrier by 14.3 kcal mol^−1^ relative to the same reaction in aqueous solution. Taking into account the deprotonation of noradrenaline prior to the hydride transfer reaction, the activation barrier in the enzyme is calculated to be 20.3 ± 1.6 kcal mol^−1^, being in reasonably good agreement with the correlated experimental value of 16.3 kcal mol^−1^ (O'Carroll et al., 1986). The results presented here offer a strong support that both MAO A and MAO B isoforms function by the same hydride transfer mechanism.

In this study we also calculated the effects of point mutations on the activation free energy. The choice of investigated systems was prompted by the experimental work of Edmondson and co-workers (Husain et al., 1982; Li et al., 2006). Our results show that the p*K*_a_ uncorrected values for the activation free energies are as follows (in kcal mol^−1^): WT MAO A 18.7, Tyr444Phe 18.2, Tyr444Leu 19.3, Tyr444Trp, and Tyr444His 25.7. For the last mutant, it is essential that His444 is neutral, otherwise in monoprotonated His444 the barrier raises for additional 5 kcal mol^−1^. Taken all together, our analysis confirmed the functional importance of the probed tyrosine residue, since mutations to Leu, Trp, and His produced enzymes which are less efficient, culminating with the Tyr444His mutant that is five orders of magnitude a slower enzyme than WT. Interestingly, Tyr444Phe mutation even slightly lowered the activation free-energy, which strongly suggests that the role of the probed Tyr44 residue is predominantly exerted through its aromatic moiety, rather than through the hydroxyl –OH group, which is a significant observation. It follows that the efficiency of the wild-type MAO A enzyme and its Tyr444 mutants is WT = Tyr444Phe > Tyr444Leu > Tyr444Trp > Tyr444His, and it could be qualitatively related to the mentioned experimental results in MAO B (Husain et al., 1982; Li et al., 2006), which read WT > Tyr444Phe > Tyr444His≈Tyr444Leu > Tyr444Trp. Another very interesting aspect is provided with His and Glu mutants regarding their protonation forms. It turns out that Tyr444His(0) mutant, where His residue is neutral, gives an enzyme with 7 kcal mol^−1^ higher activation free-energy than WT, which is even further increased by another 9 kcal mol^−1^ to Δ*G*^‡^ = 34.9 kcal mol^−1^ in Tyr444His(+) upon protonating the histidine residue, thus even exceeding the value for the aqueous solution. This observation is consistent with the idea of a positive charge build-up on the substrate in the transition state, thus strongly confirming the anionic nature of the abstracted hydride H^−^. Therefore, positively charged species near the active site have an anti-catalytic effect as clearly indicated by our results. To further test this concept, we investigated two additional Tyr444Glu mutants corresponding to unionized and negatively charged glutamic acid residues. For the neutral form, Tyr444Glu(0), the barrier is higher than for the WT, assuming Δ*G*^‡^ = 23.8 kcal mol^−1^, but is significantly reduced to Δ*G*^‡^ = 21.7 kcal mol^−1^, when Glu residue is deprotonated to –CH_2_–CH_2_–COO^−^. The induced negative charge in this active site residue stabilizes the cationic transition state, which lowers the barrier. Taken all together, the results obtained for both Tyr444His and Tyr444Glu mutants and their protonation forms provide a straightforward confirmation that MAO enzymes operate through the hydride transfer mechanism (Poberžnik et al., unpublished data).

## How important is nuclear tunneling for the MAO catalytic step?

The rate limiting step of MAO catalyzed reaction involves a hydride transfer and, as a light particle, its motion obeys the laws of quantum mechanics rather than the laws of classical mechanics. Nuclear quantum effect contributes to enzyme kinetics by vibrational zero point energy that elevates the reactant well and by tunneling through the barrier. Both effects have no classical analog and often they are refereed in the literature as “tunneling.” Both contributions give rise to the effective lowering of the barrier reflected in enlarged rate constant. Since nuclear quantum effects are mass dependent they are therefore different for H and D.

Experimentally the quantum-mechanical nature of nuclei motion in the reaction process is reflected as kinetic isotope effect (KIE) that is, by definition, the ratio of the rate constants for the species involving various isotopomers. The most pronounced are always the KIE values involving H/D isotopes, because of the large relative mass ratio of the isotopomers. Tunneling reflected in H/D KIE for MAO catalyzed reactions was addressed by several experimental studies. Husain et al. (1982) studied bovine liver MAO B catalyzed decomposition of benzylamine. The kinetic parameters were deduced from UV/VIS detection of an aldehyde signal as a function of time at 25°C. The H/D KIE was dependent on the oxygen level. For the oxygen and benzylamine saturation case the observed H/D KIE was 6.4–6.7, while at low oxygen levels the value increased to 8.7. In a detailed study of Walker and Edmondson (Walker and Edmondson, 1994) the authors applied advanced data processing techniques to address a series of *para* and *meta* substituted benzylamine analogs. The H/D KIE value for unsubstituted benzylamine was between 8.2 and 10.1, depending on the level of oxygen. For the other substituted benzylamine species the H/D KIE values were between 6.5 for *p*-Br substitution and 14.1 for *m*-Cl substitution. Tunneling in recombinant human liver MAO A catalyzed decomposition of *para*-substituted benzylamines was studied by Miller and Edmondson (1999a) and the reported values range between 6 and 13. Scrutton and co-workers studied pH dependence of KIE in recombinant human liver MAO A catalyzed decomposition of benzylamine and the value decreases from ~13 to 8 with increasing pH value (Dunn et al., 2008). Wang and Edmondson addressed tunneling in a rat MAO A for a series of *p*-substituted benzylamines (Walker and Edmondson, 1994; Wang and Edmondson, 2011), concluding that H/D KIE values are pH–independent and range from 7 to 14, demonstrating a rate-limiting α–CH bond cleavage step in catalysis.

Interesting proposals were given for the role of tunneling and dynamical effects in enzyme catalysis. These proposals were mainly based on experimental detection and careful quantization of hydrogen (radical, proton, and hydride) tunneling in enzymatic reactions. Several independent groups gathered evidence that room temperature nuclear tunneling occurs in several enzymatic reaction, especially those involving C–H bond activation (Hay et al., 2009; Klinman, 2009, 2013; Klinman and Kohen, 2013). The donor-acceptor distance played a special role in these proposals on which depends the shape of the potential for hydrogen transfer. It was long ago proposed that steric strain and compression can help catalysis (Jencks, 1987). The rationale behind assigning special relevance for catalysis to the modes involving donor-acceptor distance is that the authors suggest that for the compressed case the barrier for hydrogen transfer just gets narrower at the preserved height, which is a prerequisite for tunneling. The experimental results were interpreted by the vibronic formula approach (Klinman, 2013; Klinman and Kohen, 2013) and by a theory of electron transfer-coupled hydrogen transfer (Hammes-Schiffer, 2010). In particular, recent studies argued that the promoting mode proposal is consistent with pressure effects on enzymatic reactions, and that the observed pressure effects support the idea of vibrationally enhanced catalysis. By critical recompilation of the experimental and multiscale simulation data we have demonstrated that serious inconsistencies exist in the evidence to support these hypotheses (Kamerlin et al., 2010). Tunneling reflected in H/D KIE decreases upon compression, and external pressure does not lead to the applicable compression of the free energy surface. Moreover, pressure experiments do not provide actual evidence for vibrationally enhanced catalysis (Hay et al., 2007). Finally, the temperature dependence of the entropy change in hydrogen transfer reactions is shown to reflect simple electrostatic effects (Liu and Warshel, 2007). Hydrogen transfer reactions in enzymology can be explained by the transition state theory and the concept of promoting modes is not necessary. It is enough to know the probability density for the reactive system at the transition state and at the equilibrium. Speaking about the computational methods to calculate the KIE, the method of choice is path integration, where each quantum atoms is represented by a necklace of beads. The method yields correct ensemble averages and probability densities are different for H and D giving rise to different activation free energies. Tunneling is not a dynamical phenomenon and technically speaking KIE can be calculated also by Monte Carlo method.

In this section we report the results of the path integration calculated H/D KIE of the rate-limiting step of dopamine decomposition catalyzed by MAO B (Mavri et al., 2016). We would like to reiterate that the experimental data for dopamine are not available, but for structurally closely related substituted benzylamines and MAO B the values are between 6.5 and 14.1 indicating sensitivity of tunneling to all atomic details of the reactive system. We decided to proceed with dopamine, because of its immense importance in neuroscience and because we have developed simulation protocol for this reaction for classical treatment of nuclear motion (Repić et al., 2014b). In our study, we considered the full dimensionality of the enzyme. Path integration was implemented in the form of quantum classical path (QCP) method with an EVB potential energy surface. We obtained classical EVB reaction profile of the reacting system and its surrounding protein with water molecules by the procedure described above. The QCP approach is based on the isomorphism between the nuclear wavefunction involving all the vibrational levels and the ring of quasiparticles. As the temperatures are approaching zero, the quantum correction to free energy reduces to the contribution of the zeroth vibrational level and matches the zero point energy. At the finite temperature values contribution from all the excited states are included. Please note that the harmonic force constants connection quasiparticles are larger for D than for H and the H necklace is, therefore, more delocalized.

In order to evaluate the QCP correction to the activation free energy, it is necessary to perform simulation for the transition state and the reactant minimum. Both simulations are performed for H and D, respectively. Since the wave function is more delocalized for hydrogen than for deuterium, the corresponding probability densities and therewith associated free energy values are different. The procedure was as follows. Initially, we used exactly the same protocol as for calculation of the reaction profile with the classical treatment of nuclear motion. Equilibration of 600 ps proved to be sufficient and was performed for the system constrained with reaction coordinate corresponding to the reactant well and to the transition state. We quantized nuclear motion for the dopamine methylene group next to the amino group and the N5 atom of the flavin moiety using 18 beads. In this way, the motion of four atoms was quantized corresponding to 12 degrees of freedom. 100 ps of QCP simulations followed for both H and D. When performing calculations for the D isotopomer, both hydrogen atoms of the methylene group were replaced by D in order to facilitate comparison with the experiment. The error was estimated by using 8 different starting points that were 1 ps apart. Our computational strategy evaluated the relevant nuclear quantum corrections and gave the observed kinetic isotope effect of 12.8 ± 0.3 what is in agreement with the available experimental values in the range 6.5–14.1 for MAO B catalyzed decomposition of substituted benzylamines. The latter represent structurally the closest analogs of dopamine for which H/D KIE was determined. The calculated H/D KIE gives additional piece of evidence that the proposed hydride mechanism is valid. The H/D KIE values for the enzymes where the contribution to the rate constant comes only from the zero point energy of the reactant well are between 3 and 8. The elevated value for MAO B indicates that non-negligible contribution comes from tunneling through the barrier in the reaction coordinate region around the transition state. The calculated H/D KIE can be compared with the work of Kästner and co-workers for benzylamine decomposition catalyzed by MAO B (Zenn et al., 2015), who calculated H/D KIE by diagonalizing mass weighted Hessian for reactant well and transition state, respectively, by considering DFT described reactive QM part of the system. Zero point energy corrections were calculated for both isotopomers. The “through the barriers” contribution to tunneling was calculated by one-dimensional Eckart model allowing for the analytical solution. For the lowest barrier conformation the authors obtained a H/D KIE value of 3.5, while for three others conformations the values 2.4, 2.7, and 3.7 were reported. We feel that these H/D KIE values cluster around the lower limit, since they do not include anharmonicities of all other vibrational modes but the CH stretching.

Our calculations once more demonstrated that the applied rigorous QCP approach is a reliable computational tool and it allows for quantum mechanical contributions to activation free energies even in the case when significant tunneling contributions to the rate constant are present. It is worth to comment about the meaning of the H/D kinetic isotope effect for the catalytic decomposition of dopamine. The fact is that H isotopomer is decomposed over twelve times faster than the corresponding D isotopomer. Since very much the same effect is expected in the corresponding reaction in aqueous solution the contribution to catalytic effect is highly probable expected to be zero. This phenomenon was observed for lypoxygenase with a spectacular H/D KIE of 81. It remains a challenge to perform the isotope resolved kinetic experiments for the reaction between dopamine and lumiflavin in aqueous solution. Because of very low reaction rate this is a very demanding task.

Studies of H/D KIE have, beside insight to the enzyme mechanism, relevance in the field of deuterated drugs. For drug design and pharmacokinetics H/D KIE are highly relevant in the context of advent of D substituted drug D isotopomers (Katsnelson, 2013). In the present case treatment of patients with deuterated L-dopa would result in a prolonged clearance time.

## MAO, reactive oxygen species, and neurodegeneration

Neurodegenerative diseases are mainly caused by oxidative stress. Among other sources, MAO catalyzed oxidative deamination reactions produce hydrogen peroxide as a by-product giving rise to several reactive oxygen species (ROS) that are responsible for oxidative stress. For example, H_2_O_2_ molecules easily undergo Fenton-type chemistry to give OH^•^ radicals. A gross scheme of neurodegeneration on the molecular level is based on two pathways. Firstly, reactive species oxidize heavy atom ions, which enhances the interaction with α–synuclein (αSyn), thus promoting its folding to the beta form and giving rise to insoluble amyloid plaques. The latter prevents the function of vesicular transport leading to gradual neuronal death. In the second pathway, radical species, OH^•^ in particular, react with the methylene groups of the apolar part of the lipid bilayer of either the cell or mitochondrial wall, resulting in membrane leakage followed by dyshomeostasis, loss of resting potential and neuron death. Inflammation that follows is additional rich source of ROS. Epidemiological data show that the incidence of neurodegenerative diseases rapidly increases with the age. MAO inhibition is an important strategy for the prevention and treatment of neurodegenerative diseases. MAO is not the only source of ROS, electron transfer (ET) chain is even a richer source. It is worth to stress that ET cannot be inhibited, while MAO inhibition is possible. ROS originating from inflammation can be avoided to significant extents by COX–2 inhibition. In this section we will give an overview of the processes associated with these reactions that can be employed in developing strategies for the prevention and treatment of neurodegeneration. For a very recent review the reader should refer to reference (Pavlin et al., 2016).

The central nervous system (CNS) is vulnerable to oxidative stress and neurodegeneration is its direct consequence. An important reason why central nervous system is so prone to oxidative stress is that human brain consumes about 10 times more oxygen than is the average over all other tissues, which is directly linked to the high-energy consumption of neural signal transduction. Human brain represents about 2% of the body weight and consumes 20% of the oxygen. In addition, neurons have large surface to volume ratio and therefore there is higher probability for the cellular membrane damage than for other cells. In contrast to other cells, neurons are non-replicating and the brain is, despite massive redundancy, sensitive to the loss of function if too many neurons die. Neurodegeneration is a very complex pathological process. We are still very far from understanding all the details, particularly on a molecular level. Nevertheless, ROS, arising from several sources, including the non-enzymatic oxidation of dopamine, electron transfer chain, molecular oxygen, MAO catalyzed metabolism of biogenic and dietary amines, and inflammatory processes, are largely responsible for the damage to neurons. Additional complication is that initial damage of neurons triggers inflammatory process response that produces even more ROS resulting in perpetual damages of the neurons. ROS can harm the membranes of either neurons or mitochondria, thus inducing spillage of the contents, depolarization and loss of function, followed by fast neuronal death. Possibly more important than the toxic mechanism of ROS, is the heavy metal ion mediated one, in which the metal ions, especially copper and iron are oxidized, enhancing their binding to αSyn that then folds to a significantly less soluble beta form, which further aggregates into amyloid plaques. Keeping reasonably low levels of ROS is essential for homeostasis, while complete removal of ROS would have deleterious effect on the immune system. Glutathione (GSH), present at milimolar concentrations in most cell types, is a major intracellular reducing agent owing to its cysteine thiol group. Heavy metal ions form stable complexes with GSH, thus significantly decreasing its scavenging potential, and are involved in the Fenton and Haber–Weiss reactions producing OH^•^. On the other hand, H_2_O_2_ production from the non-enzymatic metal ions mediated oxidative deamination of dopamine and noradrenaline seems to be particularly problematic, since it is not restricted to the mitochondrial membrane, where H_2_O_2_ decomposing enzymes, such as catalase and glutathione peroxidase are located in significant quantities. These are possible explanations for the involvement of dopaminergic neurons in the initial stages of Parkinson's disease, while, at the same time, the loss of olfactory neurons, which are mainly glutaminergic, gives strong evidence for the essential role of either the electron transfer chain or inflammatory processes in neurodegeneration.

There are many dietary components that can not only scavenge ROS but also influence some of the biochemical events (signal transduction, stress protein synthesis, glycation, and toxin generation) associated with neurodegenerative pathologies, thereby either ameliorating the risks or slowing down the progression of the disease. In general, food containing sulfur rich compounds, such as garlic, onion, and avocados, are also good options. Bilirubin is a very efficient ROS scavenger (Joshi et al., 1995), which provides a possible explanation for a low incidence of cardiovascular diseases and, to a certain extent, neurodegeneration in patients with Gilbert–Meulengracht syndrome. A promising strategy for the prevention and, to a certain extent, treatment of neurodegeneration is the administration of curcumin, an essential ingredient of curry, which has recently been demonstrated to have significant neuroprotective potential (Trujillo et al., 2014). Interestingly, the prevalence of Alzheimer's disease in India among adults aged between 70 and 79 years is 4.4 times lower than in the USA (Mishra and Palanivelu, 2008). Green tea and coffee drinking seems to have neuroprotective potential. Catechins found in green tea can penetrate the hematoencephalic barrier and they act as metal chelating agents and ROS scavengers.

The ROS production originating from inflammatory processes in the central neural system can be blocked at the level of the arachidonic acid cascade with one of the COX–2 selective non-steroidal anti-inflammatory drugs (NSAID). Ibuprofen seems to be a first-choice, because of its low ulceration potential (Gao et al., 2011). However, it remains a challenge to balance the benefit of NSAID administration with its unwanted side effects and to give recommendations for administering NSAID in the context of neuroprotection. It is probably not recommendable to administer NSAID to patients with no signs of neurodegenerative diseases, particularly as long-term therapies.

One can lower ROS production through the MAO pathway by inhibiting MAO with one of the irreversible MAO B inhibitors, such as selegiline and rasagiline. We suggested chemical mechanism of the inhibition reaction (Borštnar et al., 2011). MAO A inhibitors, such as clorgyline, seem to be less appropriate because of their psychoactive properties (Pavlin et al., 2013). An unknown substance(s) in tobacco smoke also irreversibly inhibit MAO by up to 60%, suggesting that sporadic smoking in low quantities, e.g., one cigar per week, could be beneficial for the prevention of neurodegeneration (Fowler et al., 1996), while balancing its potential in the development of neoplasia and cardiovascular diseases. Interestingly, nicotine *per se* is a reversible inhibitor of MAO (Pavlin and Sket, 1993), while its metabolite nornicotine binds to the arginine side chain in αSyn, thus preventing conformational change to its β–form (Dickerson and Janda, 2003). Novel, promising strategies for design of drugs used in treatment of neurodegeneration are appearing. An interesting strategy is development of multi-target drugs possessing both cholinesterase and MAO-inhibitory activity like Ladostygil (Weinstock et al., 2000).

We have presented a few known chemical mechanisms of neurodegeneration on the molecular level. One can view neurodegeneration as the interplay of several chemical reactions with complex kinetics. It is still plenty of work ahead to get insight into the features of molecular events linked to neurodegeneration, paving the way toward new strategies for the prevention of and protection from these debilitating diseases.

## Conclusion and perspectives

In this article, an overview of computational methods relevant for molecular modeling of the processes relevant for central nervous system was given with a focus on simulations of enzymes, receptors, transporters, and reactions relevant for central nervous system. We reviewed our work concerning monomine oxidases, enzymes that catalyze the oxidative deamination of biogenic neurotransmitters and cardio- and vasoactive amines. We demonstrated by using quantum chemical methods on the cluster model level that the rate-limiting first step for monoamine oxidases represents a hydride transfer from the methylene group next to the amino moiety to the flavin N5 atom. This is followed by the substrate amino group deprotonation to the flavin N1 site, which creates a fully reduced flavin, FADH_2_, and neutral imine. Comparison between the structure and p*K*_a_ values of the ionisable groups of the active centers of MAO A and MAO B enzymes gives strong evidence that both enzymes operate by the same chemical mechanism. By using the empirical valence bond QM/MM approach, we showed that MAO B lowers the barrier of the hydride transfer reaction by 12.3 kcal mol^−1^ relative to the reference reaction in aqueous solution, corresponding to a rate-enhancement of more than 9 orders of magnitude. The barrier for the enzymatic reaction starting from the deprotonated substrate is 14.2 kcal mol^−1^. Taking into account the free energy cost of dopamine deprotonation in the active site prior to the enzymatic reaction, the reaction barrier becomes 16.1 kcal mol^−1^, being in excellent agreement with the available experimental value of 16.5 kcal mol^−1^. Assuming hydride transfer, we calculated H/D kinetic isotope effect for MAO B catalyzed decomposition of dopamine that is also in agreement with the experimental value. In conjunction with additional experimental and computational work, the data presented here improve the understanding of the mechanism of the catalytic activity of MAO, as well as a large family of flavoenzymes, which can allow for the design of novel and improved MAO B inhibitors for antiparkinsonian and neuroprotective use. Understanding and treating depression on the level of MAO A polymorphism and serotonin transporter (SERT) polymorphism is a challenge for future. Very recently published structure of SERT (Coleman et al., 2016), along with the experimental genomic data and molecular simulation of mutants represents a step forward toward the precision medicine.

## Author contributions

All authors listed, have made substantial, direct and intellectual contribution to the work, and approved it for publication.

## Funding

RV gratefully acknowledges the European Commission for an individual FP7 Marie Curie Career Integration Grant (contract number PCIG12–GA–2012–334493). CD's lab is funded by grants from The Royal Society, the Engineering and Physical Sciences Research Council (EPSRC) and the Biotechnology and Biological Sciences Research Council (BBSRC). JM thanks the Slovenian Research Agency for the financial support in the framework of Programme Group P1-0012. The authors thank COST Action CM1103 for the productive collaborations that inspired this work and for open access funding.

### Conflict of interest statement

The authors declare that the research was conducted in the absence of any commercial or financial relationships that could be construed as a potential conflict of interest.

## Abbreviations

MAO: monoamine oxidase
GPCR: G-protein coupled receptors
QM: quantum mechanics
MM: molecular mechanics
MD: molecular dynamics
QM/MM: quantum mechanical/molecular mechanical
EVB: empirical valence bond
VB: valence bond
AChE: acetylcholinesterase
BChE: butyrylcholinesterase
WT: wild type
QCP: quantum classical path
KIE: kinetic isotope effect
DFT: density functional theory
FEP: free-energy perturbation
US: umbrella sampling
CCM: cholesterol consensus motif
CRAC: cholesterol recognition/interaction amino acid
TRP: transient receptor potential
FAD: flavin adenine dinucleotide
RMSD: root-mean-square deviation
PDLP/S–LRA: semimacroscopic protein dipole/Langevin dipole approach in its linear response approximation version
DAAO: D–amino acid oxidases
TMO: tryptophan-2-monooxygenase
MTOX: *N*-methyltryptophan oxidase
CPCM: conductor-like polarizable continuum model
NBO: natural bond orbitals
SCAAS: surface-constrained all atom solvent model
FEP/US: free energy perturbation/umbrella sampling
IRC: intrinsic reaction coordinate
ROS: reactive oxygen species
αSyn: α–synuclein
ET: electron transfer
CNS: central nervous system
GSH: Glutathione
NSAID: non-steroidal anti-inflammatory drugs.
